# A Comprehensive Analysis of MicroRNAs Expressed in Susceptible and Resistant Rice Cultivars during *Rhizoctonia solani* AG1-IA Infection Causing Sheath Blight Disease

**DOI:** 10.3390/ijms21217974

**Published:** 2020-10-27

**Authors:** Ramakrishna Chopperla, Satendra K. Mangrauthia, Talluri Bhaskar Rao, Marudamuthu Balakrishnan, Sena Munuswamy Balachandran, Vellaisamy Prakasam, Gireesh Channappa

**Affiliations:** 1 ICAR-Indian Institute of Rice Research, Hyderabad 500030, India; chopperlaramakrishna@gmail.com (R.C.); talluribhaskar31@gmail.com (T.B.R.); balasena@yahoo.com (S.M.B.); vprakasam.iari@gmail.com (V.P.); giri09@gmail.com (G.C.); 2 Bioinformatics Lab, ICAR-National Academy of Agricultural Research Management, Hyderabad 500030, India; balakrishnan@naarm.org.in

**Keywords:** fungi, *Oryza sativa*, miRNA, strain, wild rice, gene expression

## Abstract

MicroRNAs regulate plant responses to fungal infections and immunity. In this study, miRNAs were identified in six rice cultivars during a *Rhizoctonia solani* Kühn AG1-IA infection using a deep sequencing approach. Known and novel miRNAs were analyzed in these rice cultivars, and a set of fungal infection/immunity-associated miRNAs and target genes were quantified by reverse transcription (RT)-qPCR in six rice cultivars. Additionally, the relative expression of these miRNAs was analyzed in different time points of the infection, wild species of rice, and in response to different strains of *R. solani*. Osa-miR1320-5p showed preferential expression during the fungal infection in all the six rice genotypes, while Osa-miR156d, Osa-miR159b, Osa-miR820c, and Osa-miR1876 were differentially regulated in susceptible and resistant genotypes. A greater degree of downregulation of miRNAs was observed during the initial time points of infection (24–72 h), suggesting a maximum molecular activity of rice-*R. solani* interaction and resistance response of the host during the early phase of infection. After *R. solani* infection, the expression of Osa-miR820c and Osa-miR156d was downregulated in *Oryza rufipogon, O. alta, O. latifolia,* and *O. minuta*, while Osa-miR397b was downregulated in all the wild rice species except *O. officinalis*. This study provided comprehensive information on the repertoire of miRNAs expressed in six sheath blight disease-susceptible and resistant *indica* and aus rice cultivars.

## 1. Introduction

Rice is the most widely consumed staple food crop of the world, especially in Asian countries. China and India hold first and second rank in total rice production (FAO: Rice Market Monitor, 2018), which ensures the food security of these two most populous nations. Being one of the most diverse crops, rice is the host of numerous insect pests and pathogens. Among the most devastative fungal pathogens of rice, *Rhizoctonia solani* causing sheath blight disease comes next to *Magnaporthe grisea* causing blast disease. The sheath blight pathogen *Rhizoctonia solani* Kühn AG1-IA (anamorph), *Thanatephorus cucumeris* (Frank) Donk (teleomorph) can cause rice yield losses in the range of 20–45% [1,2]. This is a soil-water-borne saprophytic and facultative pathogen that produces characteristic symptoms on the leaf sheath, blades, flag leaf, and panicle [3,4,5].

The management of sheath blight disease in farmer fields is still done by the application of chemical fungicides, which are hazardous to human health, cause environmental pollution, and trigger the evolution of more virulent forms of the pathogen. The tremendous progress in the identification of resistance genes and their deployment for the development of blast and bacterial blight disease-resistant rice crops [6,7,8] could not be replicated in the case of sheath blight disease. Though researchers across the globe made serious efforts to identify and map the resistance genes/quantitative trait loci (QTLs), and the development of transgenic resistance [5], commercial cultivars with desirable sheath blight resistance are still unavailable for cultivation. The critical factors responsible for the limitations to achieve sheath blight disease resistance are a lack of clear understanding of the molecular pathways and genetic elements regulating the host-pathogen interaction, pathogen biology, and polygenic and complex nature of resistance [9].

Small RNAs are critical regulators of the host defense during plant-fungal interactions [10,11]. MicroRNAs are the most studied small endogenous noncoding RNAs ubiquitously found in eukaryotic organisms [12,13]. These tiny 19–25-nucleotide-long RNAs play critical roles in post-transcriptional gene regulation by the cleavage or inhibition of the target genes [14]. The roles of miRNAs have been suggested in almost all the biological functions of plants, indicating that these are essential regulators of plant growth, metabolism, symbiotic interactions, and stress response [15,16,17,18]. The critical role of miRNAs in host-fungal interactions and resistance responses has also been studied in rice. Most of these reports are centered on *M. grisea*, a hemibiotroph fungi [7,19,20,21,22,23,24,25,26]. These studies suggest a consistent and appreciable progress in understating the role of miRNAs in rice-*M. grisea* interactions. However, efforts of similar intensity have not been made to understand miRNA-mediated genetic regulation during rice-*R. solani* interactions. It should be noted that, unlike *M. grisea*, *R. solani* is a necrotroph; hence, deciphering the expression and regulation of miRNAs of rice during *R. solani* infection will broaden the understanding of the molecular events and gene regulation during rice-fungal interactions. Recently, Wenlei et al. [27] analyzed the expression of rice miRNAs in a susceptible (Xudao 3, a japonica cultivar) and a resistant (YSBR1, a hybrid progeny of japonica/indica) rice cultivar in response to *R. solani* infection. They reported miRNAs expression after 5, 10, and 20 h of fungal inoculation. However, we did not observe a noticeable interaction between rice and *R. solani* in the first 24 h of inoculation. Basu et al. [28] reported that the initiation of *R. solani* hyphal growth and formation of infection cushions on the rice leaf surface require a minimum of 24 h after the inoculation. We performed a pilot experiment that suggested a negligible influence on miRNAs expression during the very early phase of inoculation (<24 h of inoculation). In another study, Lin et al. [29] identified 23 rice miRNAs associated with the *R. solani* defense response. Both of these reports highlight the necessity of a comprehensive approach to unravel the role of miRNAs in rice-*R. solani* interactions. Furthermore, information on miRNAs regulation during sheath blight disease in *indica* and aus rice cultivars is not yet known. The cultivated rice *Oryza sativa* has been classified into five distinct groups: *indica*, temperate japonica, tropical japonica, aus, and aromatic rice [30].

In this study, six rice genotypes, including *indica* (TN1, BPT5204, Vandana, Tetep, and Pankaj) and aus (N22) cultivars, were used to analyze the expression of rice miRNAs induced by *R. solani* AG1-IA. Among these, TN1, BPT5204, N22, and Vandana were susceptible, while Tetep and Pankaj were moderately resistant to sheath blight disease [31]. We used whole genome sequencing of small RNAs to profile the expressed miRNAs in susceptible and resistant rice cultivars. The expressed miRNAs were mapped to known sheath blight-resistant QTLs. Beside analyzing the genome-wide known and novel miRNAs in these rice cultivars, 14 of these miRNAs reported to be involved in fungal infection and immunity were quantified by reverse transcription (RT)-qPCR to decipher the fold change regulation of these miRNAs during *R. solani* infection. The target genes of these miRNAs were also quantified. The study was further expanded by analyzing the expression of a set of miRNAs induced by three different strains of *R. solani* and four different time points of infection to decipher the differential regulation of miRNAs during infection and disease establishment process. Wild rice species are reservoirs of disease-resistant genes; therefore, the most promising miRNAs were analyzed in six wild species of rice (*O. rufipogon*, *O. officinalis*, *O. alta*, *O. latifolia*, *O. minuta*, and *O. punctata*) and one African cultivated species (*O. glaberrima*). This is a most comprehensive effort to analyze the miRNAs that adds significant information on gene regulation during rice-*R. solani* interactions.

## 2. Results

### 2.1. Sequencing of Small RNAs

To identify the rice microRNAs induced by *R. solani* infection, we analyzed the small RNA sequencing data obtained from control and infected rice tissues (sheath and leaf) of susceptible (TN1, BPT5204, N22, and Vandana) and resistant (Tetep and Pankaj) genotypes. A total of ~190 million clean reads were obtained from twelve small RNA sequencing libraries after removing the adapters and low-quality sequences. Further, ~17 million clean reads of 18–24 nucleotide lengths were filtered for a downstream analysis of the miRNAs. The quality (Q30 score) of the sequencing data of all the libraries ranged from 95.61% to 96.70%, with an average of 96.44%, suggesting a reliable quality of data [32]. The details of the sequencing statistics of each library are given in Table 1.

### 2.2. Expression of Known miRNAs

**Figure 1:**
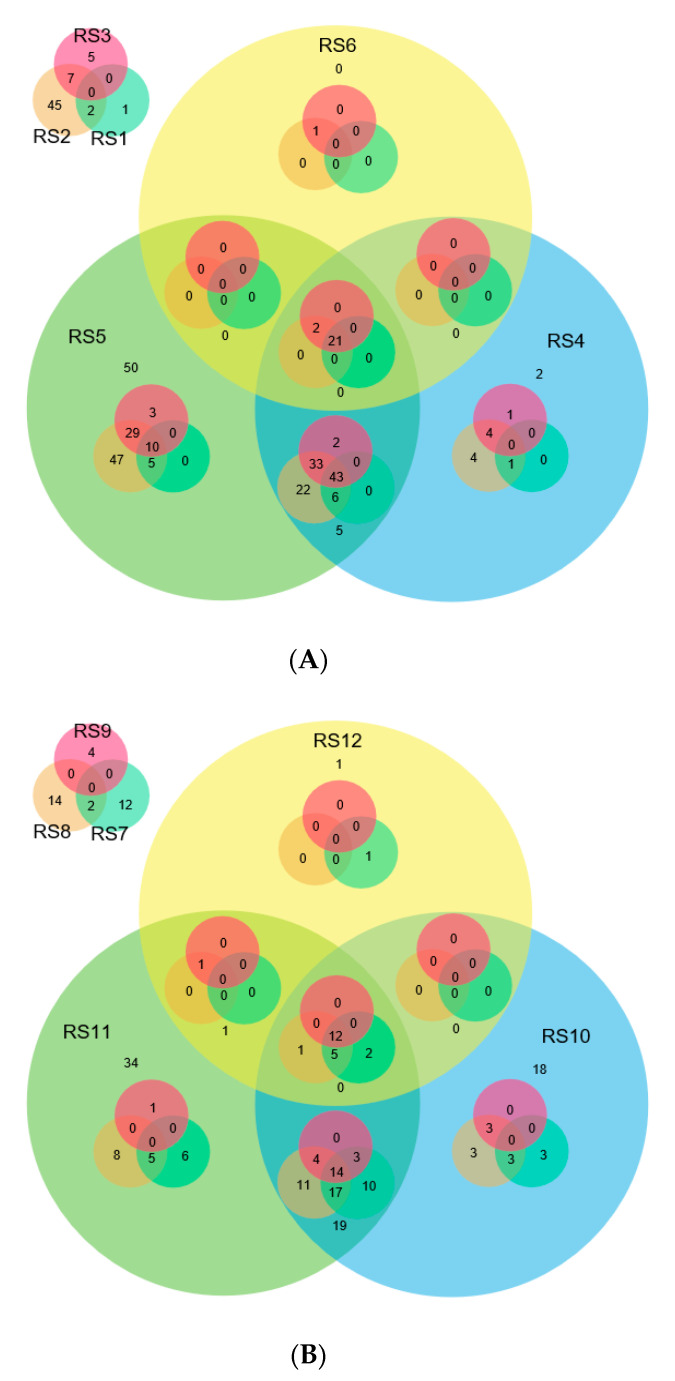
Common and exclusive known miRNAs detected in different small RNA libraries. (**A**) Venn diagram showing the common and unique miRNAs expressed under the *R. solani* infection condition. (**B**) Venn diagram showing the common and unique miRNAs under the control condition. Infected samples (RS1-TN1, RS2-BPT5204, RS3-N22, RS4-Vandana, RS5-Tetep, and RS6-Pankaj) and control samples (RS7-TN1, RS8-BPT5204, RS9-N22, RS10-Vandana, RS11-Tetep, and RS12-Pankaj).

The clean reads of small RNAs (18–24 nt) obtained after filtration were mapped to the reference rice genome, and known miRNAs were searched against the miRBase [33]. The highest number of known miRNAs (282) was identified in the infected tissue of BPT5204, whereas the lowest number (24) was identified in N22 (Table 1 and Supplementary Table S1). The most abundant miRNA families expressed in all the small RNA libraries were Osa-miR1425, Osa-miR166, Osa-miR159, Osa-miR168, Osa-miR396, Osa-miR530, and Osa-miR1862. Osa-miR1425-5p was more abundantly present in the fungal-infected libraries. Twenty-one commonly expressed known miRNAs in six *R. solani*-infected small RNA libraries were identified, whereas 12 miRNAs showed a common expression in the control libraries (Figure 1 and Supplementary Tables S2 and S3). The expression of miRNA families was analyzed across all the 12 libraries. The highest number of miRNA families was found in an infected sample of BPT5204 (137), whereas the lowest in the control sample of N22 (17). The overall observation revealed that expression of different miRNA families was greater under *R. solani* infection compared to the control condition (Supplementary Table S4).

### 2.3. Identification and Distribution of Novel miRNAs

**Figure 2:**
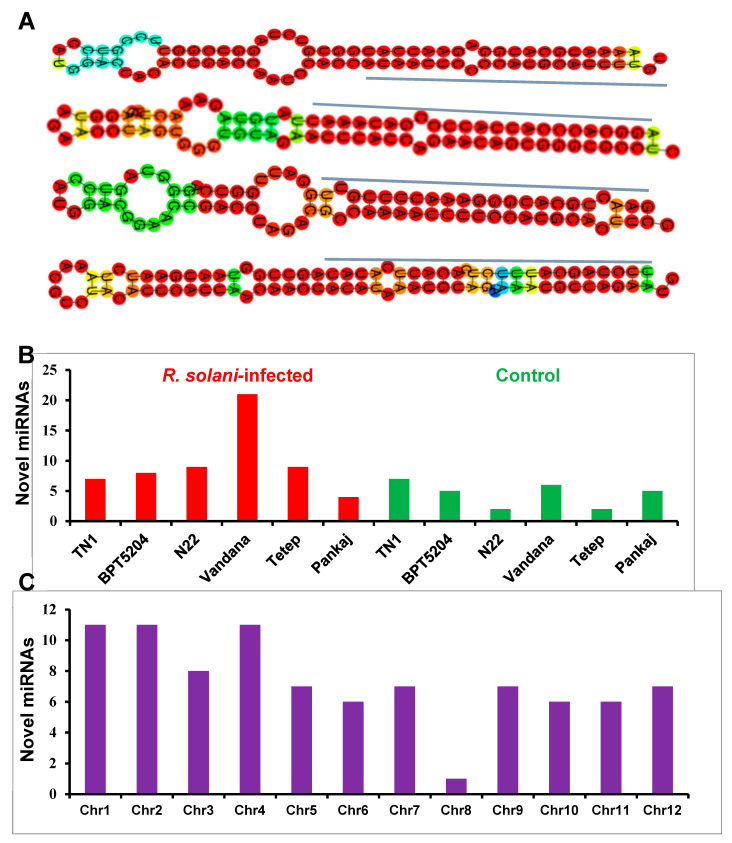
Novel miRNAs identified in small RNA libraries. (**A**) Predicted hairpin structures of precursor sequences of four novel miRNAs. The mature miRNA sequences are marked with blue color lines. (**B**) Distribution of novel miRNAs among infected (red color bars) and control (green color bars) samples. (**C**) Distribution of novel miRNAs in different chromosomes of the rice genome.

The sequencing data was used to predict 85 potential novel miRNAs from 12 small RNA sequencing libraries. The length of the consensus matured miRNA sequence of the novel miRNAs was in the range of 19 to 24 nt, of which the majority were either 21 or 24 nt (Supplementary Table S5). The secondary structure of the novel miRNAs was predicted, and few representative structures showing mature miRNAs located on the stem arms of the hairpin secondary structures are shown (Figure 2A). The number of predicted novel miRNAs was more in the *R. solani*-infected condition than the control (Figure 2B). The distribution of novel miRNAs in the rice genome showed that these were distributed across all the chromosomes. The highest number of novel miRNAs (11) was found in chromosome 1 and 4, whereas the lowest number of novel miRNAs (1) was recorded in chromosome 8 (Figure 2C).

### 2.4. Comparison of miRNAs Expression in Control and Infected Samples

**Figure 3:**
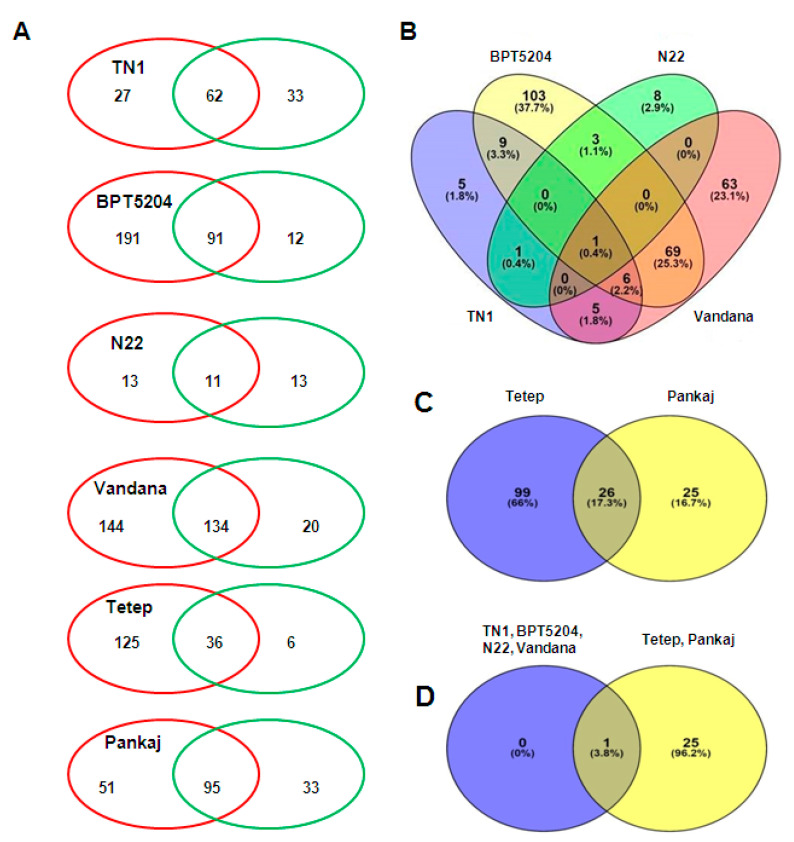
(**A**) Venn diagrams showing common and exclusively expressed miRNAs under the *R. solani*-infected and control conditions. The red color circle represents the *R. solani*-infected samples, whereas the green color circle represents the control samples. (**B**) Venn diagram of miRNAs showing exclusive expression (under *R. solani* infection) in susceptible rice cultivars. (**C**) Venn diagram of miRNAs showing exclusive expression (under *R. solani* infection) in tolerant rice cultivars. (**D**) Venn diagram of miRNAs showing exclusive expression (under *R. solani* infection) in tolerant and susceptible rice cultivars.

The miRNAs expressed in the control and fungal infected tissues were compared for each of the six genotypes. The preferentially expressed miRNAs under the control and infected conditions were categorized along with the commonly expressed miRNAs (Figure 3A and Supplementary Table S6). In general, miRNAs showing preferential expression in the infected tissue were greater in number as compared to miRNAs expressed preferentially in the control tissue. These preferentially expressed miRNAs under infected conditions were compared to identify the common miRNAs among the susceptible genotypes. The resultant Venn diagram revealed that Osa-miR1320-5p was the preferentially expressed (under the fungal-infected condition) common miRNA in all the susceptible genotypes (Figure 3B and Supplementary Table S7). Similarly, 26 common miRNAs showed preferential expression in fungal-infected-resistant genotypes (Figure 3C and Supplementary Table S8). Notably, Osa-miR1320-5p was preferentially expressed in common miRNA in all the six genotypes (susceptible and resistant) under the fungal-infected condition (Figure 3D and Supplementary Table S9).

### 2.5. Differential Regulation of miRNAs and Target Genes

**Figure 4:**
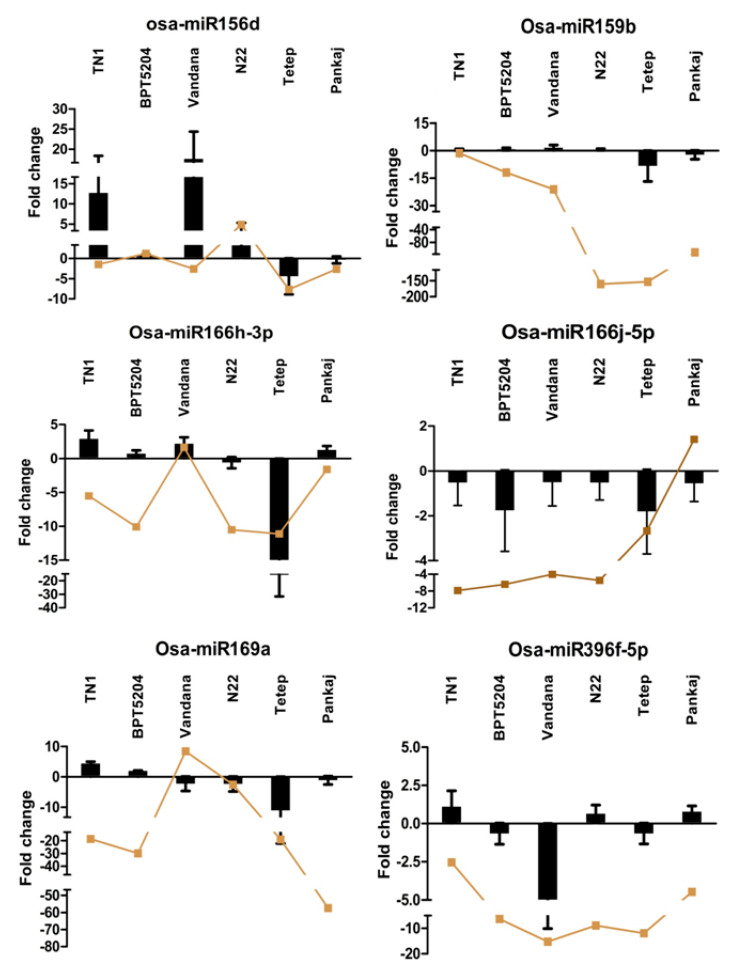

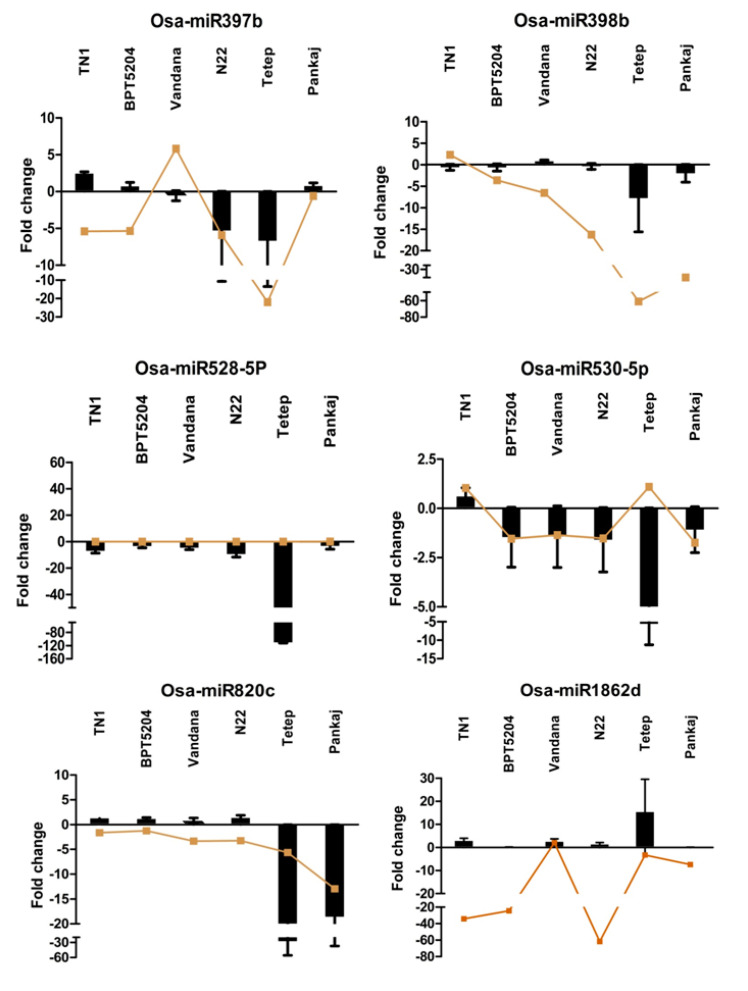

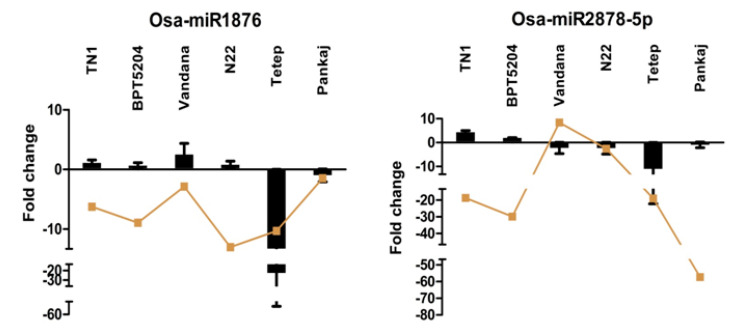
Expression analysis of miRNAs and their target genes. miRNAs and their respective target genes expressing under *R. solani* infection were quantitatively analyzed through RT-qPCR. Data represented after internal reference genes U6 (for miRNAs) and *OsActin1* (for target genes) normalization. The error bars represent the standard error of three biological replicates. *X*-axis: samples and *Y*-axis: fold change expression in the fungal-infected sample in comparison to the control. Bars represent miRNAs, while lines represent target genes expression.

Among the commonly expressed miRNAs under fungal infection, 14 miRNAs i.e., Osa-miR156d, Osa-miR159b, Osa-miR166h-3p, Osa-miR166j-5p, Osa-miR169a, Osa-miR396f-5p, Osa-miR397b, Osa-miR398b, Osa-miR528-5p, Osa-miR530-5p, Osa-miR820c, Osa-miR1862d, Osa-miR1876, and Osa-miR2878-5p, were analyzed for their expression levels through RT-qPCR. The target genes of these miRNAs, such as SLP16, OsMYB5, similar to subtilisin-like protease, similar to HAP2 subunit of the HAP complex, similar to laccase, F-box component of the SKP-Cullin-F box (SCF) E3 ubiquitin ligase complex, No Apical Meristem (NAM) protein domain containing protein, 2OG-Fe (II) oxygenase family protein, nucleus-encoded chloroplast protein, pentatricopeptide repeat domain containing protein, similar to RNA binding motif protein, transcriptional activator/repressor, and similar to homeobox-leucine zipper protein HOX9, were predicted (Supplementary Table S10). Fold change expression of the miRNAs and target genes in the infected tissue was calculated by comparing their expression levels under the control condition. All the miRNAs except Osa-miR1862d showed downregulation in the resistant genotype Tetep under the fungal-infected condition. Similarly, resistant genotype Pankaj showed a downregulation of all the miRNAs except Osa-miR166h-3p, Osa-miR396f-5p, Osa-miR397b, and Osa-miR1862d. Notably, Osa-miR1862d showed upregulation, while Osa-miR166j-5p and Osa-miR528-5p showed downregulation in all the six genotypes under *R. solani* infection. The miRNAs such as Osa-miR156d, Osa-miR159b, Osa-miR820c, and Osa-miR1876 showed a distinct opposite expression pattern between susceptible and resistant genotypes, i.e., upregulation in susceptible and downregulation in resistant genotypes (Figure 4). Expression analysis of the 14 target genes of all these miRNAs was analyzed in the same samples that were used for the miRNAs expression analysis. Generally, the regulation of the target genes expression was opposite to the miRNAs expression (Figure 4). Target gene of Osa-miR169a (similar to HAP2 subunit of the HAP complex) showed a 45-fold-induced expression in resistant cultivar Tetep. Similarly, the target gene of Osa-miR166j-5p (similar to subtilisin-like protease) showed upregulation in Pankaj but downregulation in other rice cultivars. The target gene of Osa-miR528-5p (F-box component of the SKP-Cullin-F box (SCF) E3 ubiquitin ligase complex) showed upregulation in both the resistant cultivars but downregulation in all four susceptible cultivars.

### 2.6. Expression Analysis of miRNAs at Different Time Points of Infection

**Figure 5:**
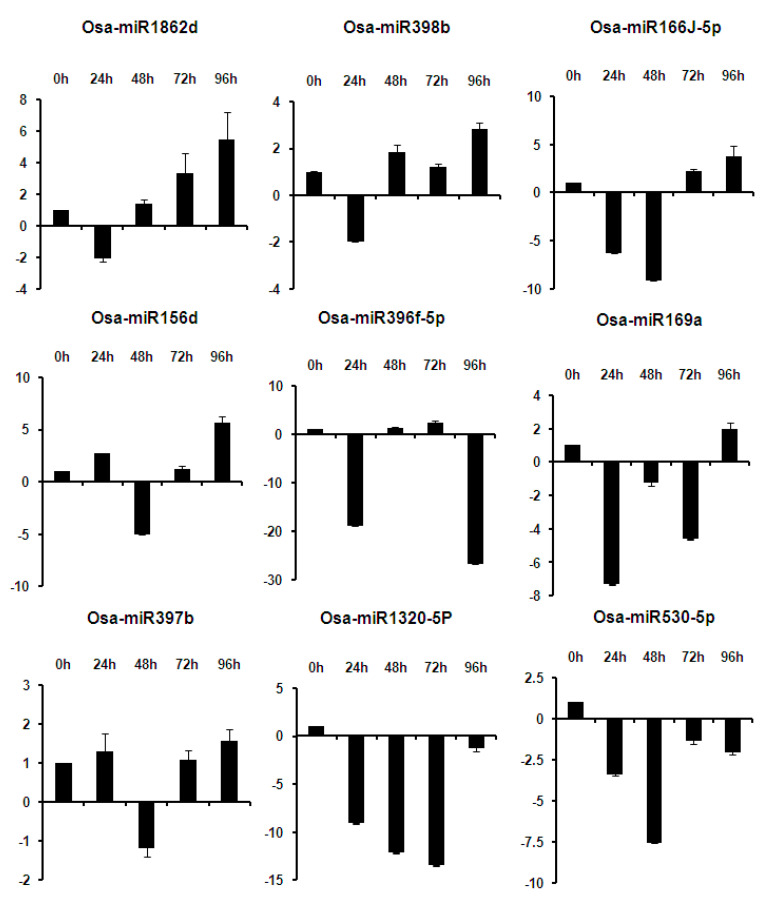

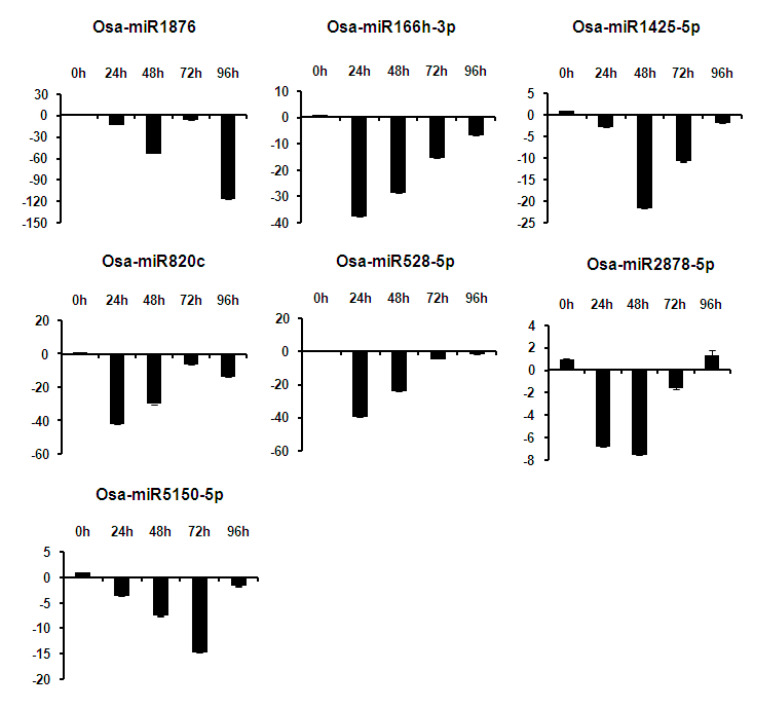
RT-qPCR expression analysis of miRNAs in the TN1 genotype at different time points after *R. solani* inoculation. Data is represented after internal reference gene U6 normalization. The error bars represent the standard error of three biological replicates. *X*-axis: samples and *Y*-axis: relative expression of miRNAs.

The miRNAs regulation during different time points of fungal infection was analyzed in TN1 at 0 h, 24 h, 48 h, 72 h, and 96 h after *R. solani* AG1 IA strain Wgl-2 inoculation. Osa-miR1320-5p, Osa-miR530-5p, Osa-miR1876, Osa-miR166h-3p, Osa-miR1425-5p, Osa-miR820c, Osa-miR528-5p, and Osa-miR5150-5P showed downregulation during all the time points of infection, though the extent of downregulation varied. Among these, the majority showed a greater degree of downregulation during the initial phase of infection, i.e., 24–72 h after inoculation. Osa-miR1862d, Osa-miR398b, Osa-miR166j-5p, Osa-miR156d, Osa-miR169a, Osa-miR2878-5p, and Osa-miR397b showed a differential expression pattern at different time points, although the majority were downregulated during the initial time points of infection and upregulated during later time points. Specifically, Osa-miR1862d, Osa-miR398b, and Osa-miR166j-5p showed downregulation within 48h of infection but upregulation at later time points (Figure 5).

### 2.7. Expression Analysis of miRNAs Induced by Different Strains of R. solani

**Figure 6:**
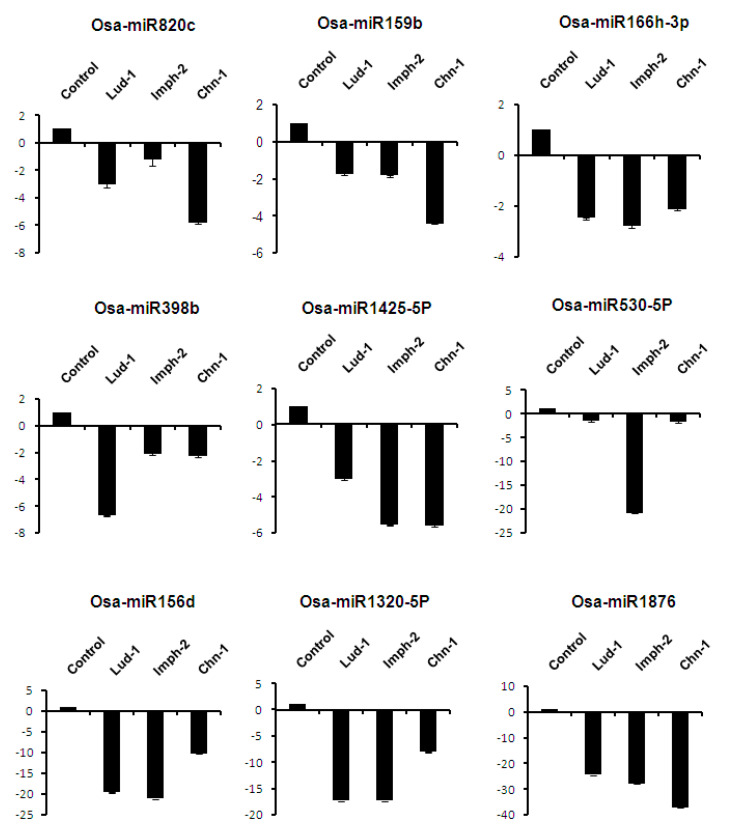

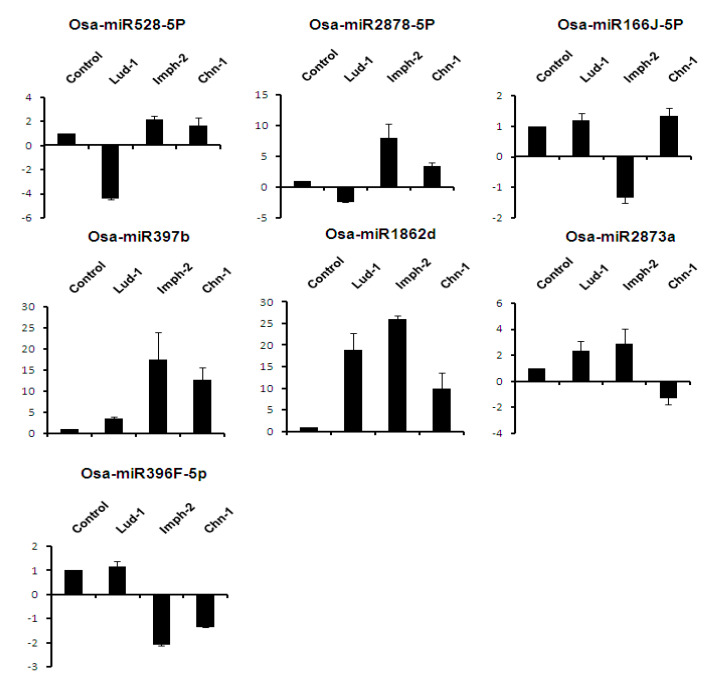
RT-qPCR expression analysis of miRNAs in the TN1 genotype inoculated with different strains of *R. solani.* Data is represented after internal reference gene U6 normalization. The error bars represent the standard error of three biological replicates. *X*-axis: samples and *Y*-axis: relative expression of miRNAs.

The fold change regulation of miRNAs was analyzed in TN1 inoculated with three different strains (one highly virulent and two moderately virulent) of *R. solani*. Nine miRNAs i.e., Osa-miR820c, Osa-miR159b, Osa-miR166h-3p, Osa-miR398b, Osa-miR1425-5P, Osa-miR530-5p, Osa-miR156d, Osa-miR1320-5P, and Osa-miR1876, showed downregulation, while Osa-miR397b and Osa-miR1862d showed upregulation during infection by all the strains of fungus. Five miRNAs, i.e., Osa-miR528-5p, Osa-miR2878-5p, Osa-miR166j-5p, Osa-miR2873a, and Osa-miR396F-5p, showed a differential expression pattern against different strains of *R. solani* (Figure 6). Osa-miR528-5p and Osa-miR2878-5p showed downregulation against the highly virulent strain Lud-1 but upregulation against the moderately virulent strains Imph-2 and Chn-1. In contrast, Osa-miR396F-5p was upregulated against Lud-1 and downregulated against the Imph-2 and Chn-1 strains.

### 2.8. Expression Analysis of R. solani Responsive miRNAs in Wild Relatives of Rice

**Figure 7:**
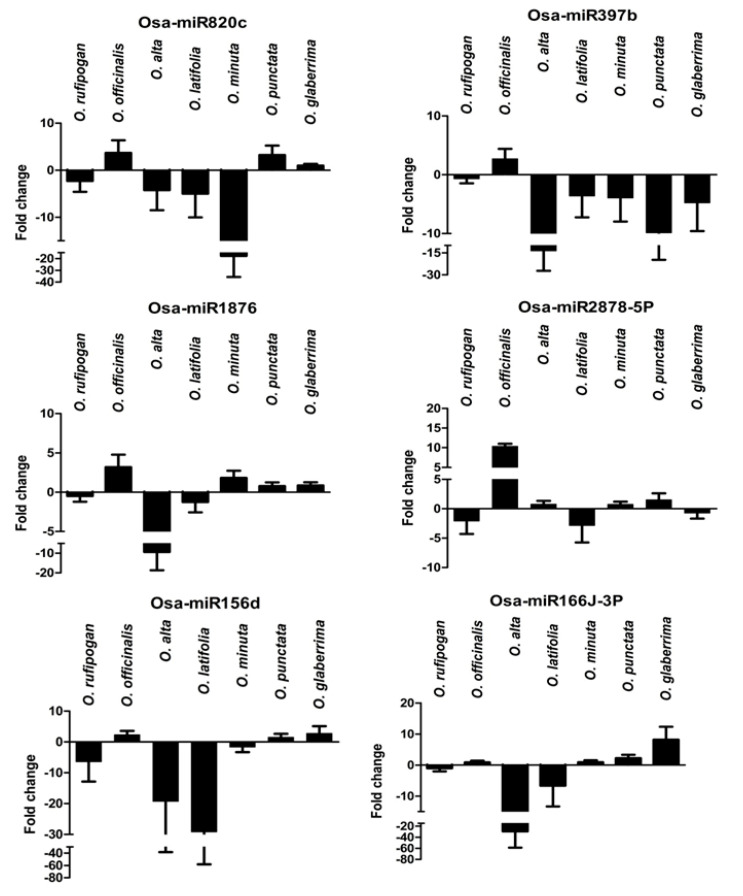
RT-qPCR expression analysis of miRNAs in wild rice species. Data is represented after internal reference gene U6 normalization. The error bars represent the standard error of three biological replicates. *X*-axis: samples and *Y*-axis: fold change expression in the fungal-infected sample in comparison to the respective controls.

Six of the *R. solani*-induced miRNAs were analyzed for their expression behavior in six different wild relatives of *O. sativa* (*O. rufipogon, O. officinalis, O. alta, O. latifolia, O. minuta,* and *O. punctata*) and in *O. glaberrima*, the second cultivated *Oryza* species grown in African countries. These species of *Oryza* were phenotyped against *R. solani* (Supplementary Table S11). The accessions of *O. rufipogon, O. officinalis, O. alta, O. latifolia,* and *O. minuta* showed a resistant phenotype (disease score 3), while *O. punctata* and *O. glaberrima* showed a moderately resistant phenotype (disease score 5). Among the six miRNAs chosen for the expression analysis, five miRNAs (Osa-miR820c, Osa-miR397b, Osa-miR1876, Osa-miR2878-5p, and Osa-miR156d) showed noticeable downregulation after fungal infection in sheath blight-resistant cultivars, specifically in Tetep. Fold change expression of these miRNAs was analyzed by comparing the expression levels in the *R. solani* inoculated sample with the control sample of each of the wild species. The miRNAs were differentially regulated in these wild species during the *R. solani* infection. Osa-miR820c and Osa-miR156d showed downregulation in *O. rufipogon, O. alta, O. latifolia,* and *O. minuta*. Osa-miR397b showed downregulation in all the wild rice species except *O. officinalis*. The *O. rufipogon*, *O. alta*, and *O. latifolia* showed downregulation of the tested miRNAs under *R. solani* infection, except Osa-miR2878-5p, which showed upregulation in *O. alta*. Notably, all the miRNAs showed upregulation in *O. officinalis* during the fungal infection. Similarly, *O. punctata* showed upregulation of all the miRNAs except Osa-miR397b. A greater degree of downregulation of Osa-miR156d and Osa-miR166j-3p in *O. alta* and *O. latifolia*, Osa-miR820c in *O. minuta,* Osa-miR397b in *O. alta* and *O. punctata,* and Osa-miR1876 in *O. alta* and upregulation of Osa-miR2878-5p in *O. officinalis* were recorded (Figure 7).

### 2.9. Mapping of R. solani-Induced miRNAs to Sheath Blight-Resistant QTL Regions

**Figure 8:**
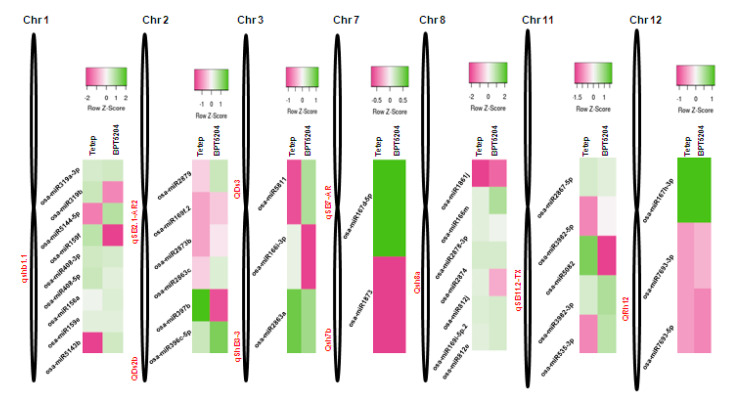
Mapping of *R*. *solani*-induced miRNAs to QTL regions conferring resistance to sheath blight disease. Heat map of these miRNAs expression in tolerant (Tetep) and susceptible (BPT5204) genotypes under the fungal infection is also shown.

The miRNAs identified in this study were mapped within or close to the reported sheath blight-resistant QTL regions located on different chromosomes of the rice genome (Supplementary Table S12). The fungal-induced miRNAs could be assigned to QTLs located on seven chromosomes (Chr1, Chr2, Chr3, Chr7, Chr8, Chr11, and Chr12). The heat map of the miRNAs lying in the QTL regions were shown by comparing their expression levels (after *R. solani* infection) in the resistant (Tetep) and susceptible (BPT 5204) genotypes. The highest number of miRNAs mapped in the sheath blight-resistant QTLs were present on Chr1 (9), followed by Chr8 (7), whereas the lowest number of miRNAs were mapped on Chr7 (2) (Figure 8). Most of these QTL-miRNAs, such as Osa-miRs 319b, 5144-5p, 159f, 5143b, 2879, 169f.2, 397b, 2863c, 5811,166i-3p, 812j, 3982-5p, 5082, and 533-3p, showed differential regulations in Tetep and BPT5204 during *R. solani* infection.

## 3. Discussion

Sheath blight disease caused by *R. solani* is a serious threat to rice cultivation worldwide. While several efforts have been made to identify the candidate genes and QTLs associated with sheath blight resistance [5,34,35], the underlying mechanisms of pathogenicity and resistance are not very well-understood [4]. MicroRNAs are well-studied key regulators of the susceptibility and resistance response of the host against fungal pathogens [21,23,36,37,38]. The discovery of miRNAs involved in plant immunity against fungal pathogens holds great potential to develop resistant cultivars by employing novel strategies [39]. The gene regulation by fungal-induced miRNAs in rice during *R. solani* infection is not yet well-understood. Recently, Wenlei et al. [27] identified miRNAs expression in rice at different time points of the *R. solani* infection (5 hpi, 10 hpi, and 20 hpi) using japonica rice cultivars. In this study, we identified and analyzed the indica and aus rice miRNAs induced by *R. solani* infection. This is a comprehensive effort for analyzing the miRNAs in six diverse indica and aus rice cultivars. Further, differential regulations of a set of identified miRNAs were analyzed during infection by different strains of *R. solani* and at different time points of infection. Unlike Wenlei et al. [27], we analyzed the expression of these miRNAs at 24 h, 48 h, 72 h, and 96 h post-inoculation. The selected resistance-associated miRNAs were analyzed in wild rice accessions that showed resistant or moderately resistant phenotypes.

Osa-miR169a, Osa-miR1425-5p, Osa-miR398b, Osa-miR2873a, Osa-miR169i-3p, Osa-miR528-5p, and Osa-miR2878-5p were commonly expressed miRNAs in all the six infected rice cultivars. Osa-miR169a, Osa-miR1425-5p, and Osa-miR398b were identified as basal response regulators, while Osa-miR2873a, Osa-miR169i-3p, Osa-miR528-5p, and Osa-miR2878-5p were identified as negative regulators involved in rice immunity against *M. oryzae* [40]. Osa-miR1425-5p was more abundantly present in *R. solani*-infected sequencing libraries. Osa-miR1425-5p and Osa-miR398b, along with the Osa-miR159b, Osa-miR396f-5p, Osa-miR530-5p, Osa-miR166j-5p, Osa-miR1862d, and Osa-miR5150-5p, showed induced expression in susceptible, as well as resistant, rice cultivars, suggesting these may be basal response regulators against the *R. solani* pathogen. Osa-miR159b was induced during the infection by rice stripe virus and *Xanthomonas oryzae oryzae* [41,42], while Osa-miR396f was induced during rice foot rot disease caused by the pathogen *Dickeya zeae* [43]. Similarly, Osa-miR530-5p showed induced expression during the rice blast infection [21]. Osa-miR1320-5p was identified as preferentially expressed miRNA during the *R. solani* infection in all the six rice cultivars. Recently, Wang et al. [24] identified Osa-miR1320-5p as the miRNA involved in rice immunity against *M. oryzae*. This can be a priority candidate miRNA for further characterization to establish its role in rice-fungal interactions.

Twenty-six common miRNAs showing preferential expression in fungal-infected resistant genotypes included Osa-miR396c-5p, Osa-miR1861i, Osa-miR1863, Osa-miR319b, Osa-miR169f, Osa-miR393a, Osa-miR393b, Osa-miR1850, Osa-miR156e, Osa-miR159c, Osa-miR167e-3p, Osa-miR167d-5p, Osa-miR167h-3p, Osa-miR535-3p, Osa-miR1857, Osa-miR2863, Osa-miR396d, and Osa-miR399j. All these miRNAs have been reported to be involved in immunity or responses against rice fungal and bacterial pathogens. The expression level of Osa-miR396c-5p was increased in a resistant rice variety, and its role was suggested in rice immunity against *M. oryzae* [26,40]. Similarly, Osa-miR1863, Osa-miR319b, Osa-miR169f, Osa-miR535-3p, Osa-miR393a, and Osa-miR396d regulate rice immunity against *M. oryzae* [22,25,40,44]. Osa-miR1850, miR393b, Osa-miR156e, Osa-miR1857, and Osa-miR2863 are regulated by fungal elicitors [19,36]. The high expression of members of the miR156, miR159, and miR167 families was reported during *M. oryzae* infection in rice [21]. Osa-miR1861i and Osa-miR2863a responded to *R. solani* infection in rice [27], while miR399 family members were responsive to arbuscular mycorrhiza fungi in maize [45]. miR156/SPL9 and miR159/GAMYB regulators play an important role in the biotic defense response in plants [46,47]. We also identified 85 potential novel miRNAs that could be involved in the sheath blight disease response or resistance and other critical functions of rice growth and metabolism. These miRNAs need to be functionally characterized using silencing or overexpression approaches [18] to fully appreciate their possible roles and biological significance during rice-*R. solani* interactions. A recent study demonstrated the crucial role of a novel miRNA (Md-miRln20) in the resistance against glomerella leaf spot disease of apples [48].

The fold change regulation of 14 miRNAs and their target genes was analyzed by RT-qPCR in six rice genotypes during *R. solani* infection. Functions of these miRNAs have been reported in fungal immunity or responses to fungal infection in rice (Supplementary Table S13). Most of these reports are from rice miRNAs induced by a hemibiotrophic fungal pathogen *M. oryzae*. However, reports on rice miRNAs induced by necrotrophic fungal *R. solani* are scarce. The resistant cultivar Pankaj showed upregulation of Osa-miR166h-3p, Osa-miR396f-5p, and Osa-miR397b during *R. solani* infection. Rice plants with increased expression of miR166k-166h and Osa-miR396f showed enhanced resistance against rice blast, bakanae, and foot rot diseases [23,25]. miR397b is a *M. oryzae* responsive miRNA [21] that regulates the lignin content through laccase genes [49]. Lignin is an integral component of the plant cell wall that is a primary target of *R. solani* during infection and disease establishment [9]. The majority of miRNAs showed downregulation in both the resistant genotypes, which included Osa-miR156d, Osa-miR159b, Osa-miR166j-5p, Osa-miR169a, Osa-miR398b, Osa-miR528-5p, Osa-miR530-5p, Osa-miR820c, and Osa-miR2878-5p. *M. oryzae* elicitor-treated leaves showed a significant reduction in Osa-miR156d accumulation [19], while infection by this pathogen led to a reduced expression of miR166j-5 [20]. miR159b is a *M. oryzae* or its elicitors responsive miRNA [7] that regulates genes associated with defense and programmed cell death, including a suite of 22 pathogenesis-related protein genes [47]. The downregulation of Osa-miR169a gives a resistant phenotype against *M. oryzae* by regulating defense-related genes and hydrogen peroxide accumulation. Osa-miR528-5p and Osa-miR2878-5p are negative regulators involved in rice immunity against *M. oryzae* [40], while Osa-miR530-5p was identified in response to *R. solani* and *M. oryzae* infection [21,29]. The upregulation of osa-miR820c was reported in response to *M. oryzae* and Rice Stripe Virus [19,50].

In contrast to the downregulation of miR398b in resistant genotypes during the *R. solani* infection observed in this study, the overexpression of miR398b boosted the resistance against *M. oryzae* by enhancing the hydrogen peroxide (H_2_O_2_) accumulation [51]. This could be due to the contrast nature of the pathogens. Necrotrophic *R. solani* prefers to surviving on dead tissue; therefore, a reduction of reactive oxygen species (ROS) accumulation could be one strategy of plants to minimize cell death and the growth of fungus, while biotroph *M. oryzae* spread can be stopped by a hypersensitive response and programmed cell death by enhancing the accumulation of ROS at the site of infection. It is desirable to decipher the network of the miR169a-ROS-miR398b gene regulation model in the rice-fungal interaction. Osa-miR1862d showed an induced expression in all the six genotypes after *R. solani* infection. This miRNA is responsive to *M. oryzae* or its elicitors [25]. Osa-miR156d, Osa-miR159b, Osa-miR820c, and Osa-miR1876 are important candidates for the further characterization of their role in sheath blight resistance, as they showed a contrasting expression pattern between susceptible and resistant rice cultivars. While analyzing the target genes in infected rice samples, a subtilisin-like protease showed upregulation in the resistant cultivar Pankaj but downregulation in other rice cultivars. The subtilisin-like protease showed antifungal activity against a broad range of fungi, including *R. solani*, *Fusarium oxysporum*, *Cytospora chrysosperma*, *Alternaria alternate*, and *Sclerotinia sclerotiorum* [52]. Similarly, the F-box component of the SKP-Cullin-F box (SCF) E3 ubiquitin ligase complex showed upregulation in both the resistant cultivars but downregulation in the susceptible rice cultivars. Jasmonic acid (JA) is a key regulator of the host defense pathway. A core JA signaling framework includes F-box protein, which forms the functional SCF E3 ubiquitin ligase complex [53,54]. MicroRNAs such as 319b, 159f, 169f.2, 397b, 2863c, and 166i-3p showing differential regulation in susceptible and resistant cultivars were mapped within the sheath blight-resistant QTLs. These miRNAs have been reported to play an important role in the antifungal response of the host. Along with these miRNAs, a few more miRNAs showing differential regulation were mapped in the sheath blight-resistant QTLs (Figure 8). Further probing of the function of these miRNAs would help in the molecular analysis of the QTL-guided phenotypes.

We analyzed the fold change regulation of miRNAs against different strains of *R. solani*. The majority of miRNAs showed a similar expression pattern, suggesting that the regulation of miRNAs is not much influenced by the strains of *R. solani* infecting rice. Notably, Osa-miR528-5p, Osa-miR2878-5p, Osa-miR166j-5p, Osa-miR2873a, and Osa-miR396F-5p showed differential regulation, suggesting that these miRNAs might be involved in strain-specific host-pathogen interactions. Further characterization of these miRNAs may shed light on their specific roles during the sheath blight disease of rice. The fold change expression of miRNAs was also analyzed at different time points of infection. The expression of Osa-miR1320-5p, Osa-miR530-5p, Osa-miR1876, Osa-miR166h-3p, Osa-miR1425-5p, Osa-miR820c, Osa-miR528-5p, and Osa-miR5150-5P was reduced during all the time points of infection, suggesting the critical role of these miRNAs in rice responses against *R. solani.* These miRNAs might be regulating key pathways involved in the sustained antifungal response. The expression of Osa-miR1862d, Osa-miR398b, Osa-miR166j-5p, Osa-miR156d, Osa-miR169a, Osa-miR2878-5p, and Osa-miR397b was differentially regulated at different time points, suggesting that the host may recruit a different set of miRNAs as the disease progresses. Further characterization of these miRNAs might help in understanding the host response and molecular events during the disease progression and establishment. The greater degree of downregulation of these miRNAs during the initial stage of infection indicates the maximum activity of the host-pathogen interaction within 72 h of infection. We had a similar observation while analyzing the expression of genes encoding pectin-degrading enzymes of *R. solani*. Maximum induction of the fungal genes was observed within 72 h of infection [55].

We analyzed the expression profiles of six *R. solani* defense-associated miRNAs from six wild species of rice and a cultivated African rice *O. glaberrima*. The information on miRNAs expression in wild rice is very scarce. Only a few reports showed their expression and roles in abiotic stresses [36,56,57,58]. However, the reports of miRNAs expression in wild species of rice under diseased conditions, and specifically during *R. solani* infection, are missing. In view of their hardy nature and being the source of many resistance genes, we analyzed the expression of the six most promising miRNAs in these seven Oryza species. *O. rufipogon*, *O. alta*, and *O. latifolia* showed the downregulation of defense-associated miRNAs suggesting, their important role in *R. solani* resistance phenotypes. These wild species of rice showed a resistance phenotype with a disease score of 3.0 (Supplementary Table S11). *O. rufipogon, O. alta, O. latifolia,* and *O. minuta* showed downregulation of Osa-miR820c and Osa-miR156d, which have been shown to play an important role in *Rice-Magnaporthe* interactions (Supplementary Table S13). After the *R. solani* infection, Osa-miR397b showed downregulation in five wild rice species and *O. glaberrima*. This particular miRNA was shown to play an important role in rice domestication [59]. It would be interesting to decipher the gene regulation network of Osa-miR397b in host-pathogen interactions vis-a-vis rice domestication. The expression analysis of *R. solani*-induced miRNAs in wild species of rice opens up a new opportunity to broaden the understanding of miRNA-mediated gene regulation during sheath blight disease. Allele mining and a deeper analysis of miRNA-encoding genes in these wild species can provide evolutionary linkages of host-pathogen interactions.

## 4. Materials and Methods

### 4.1. Plant Material, Fungal Strains, and Sample Preparation

Seeds of six rice genotypes (TN1, BPT5204, N22, Vandana, Tetep, and Pankaj) were sterilized and sown in trays. Fifteen-day-old seedlings were transplanted into mud pots (one plant per pot) and grown in a greenhouse. After 30 days of transplanting, six pots of each genotype showing similar growth and vigor were separated for further experiments. Three pots of each genotype were used for fungal inoculation, while the remaining three pots were used as the control. Plants were kept under moderate warm and moist conditions for facilitating the fungal growth. Freshly grown uniform-sized sclerotia of *R. solani* AG1 IA strain Wgl-2 were placed on leaf and sheath tissues. The infected tissue (1 cm up and down from the inoculation point) was harvested from three plants after 5 days of inoculation, and samples were pooled for RNA isolation. Similarly, samples from three control plants of each genotype were harvested. In order to analyze the expression of miRNAs at different time points, the sheath tissue of TN1 plants infected with *R. solani* AG1 IA strain Wgl-2 was harvested after 24, 48, 72 and 96 h of inoculation. Similarly, the expression of miRNAs was analyzed in response to infection by three different *R. solani* AG1-IA strains, i.e., Lud-1 (highly virulent), Imph-2 (moderately virulent), and Chn-1 (moderately virulent) [60]. TN1 plants were inoculated by these three stains, and sheath tissue was harvested for RNA isolation. Accessions of wild rice species (*O. rufipogon*, *O. officinalis*, *O. alta*, *O. latifolia*, *O. minuta*, and *O. punctata*) and cultivated African rice (*O. glaberrima*) were obtained from the plant breeding section of the Indian Institute of Rice Research, Hyderabad, India. *R. solani*-infected sheath tissue of these plants was harvested for miRNA expression analysis in wild rice species.

### 4.2. Small RNA Library Construction and Sequencing

Total RNA was isolated by Trizol reagent (Invitrogen, Carlsbad, CA, USA), as per the standard protocol. The isolated RNA was subjected to quality check (QC) and analyzed the parameters through Nanodrop, agarose gel electrophoresis, and Bioanalyzer (Agilent 2100, Palo Alto, CA, USA) for quantification, degradation, contamination, and integration. After QC, small RNA library was constructed by TruSeq Small RNA Library Preparation Kit (Illumina, San Diego, CA, USA). The final cDNA library was ready after a round of sequencing adaptor ligation, reverse transcription, PCR enrichment, purification, and size selection. The quality of the library was checked by Qubit 2.0 (testing the library concentration), Agilent 2100 (testing the insert size), and Q-PCR (quantification of the library effective concentration). The qualified libraries were fed into a HiSeq 2500 sequencer according to its effective concentration and expected data volume.

### 4.3. Sequence Analysis

**Figure 9:**
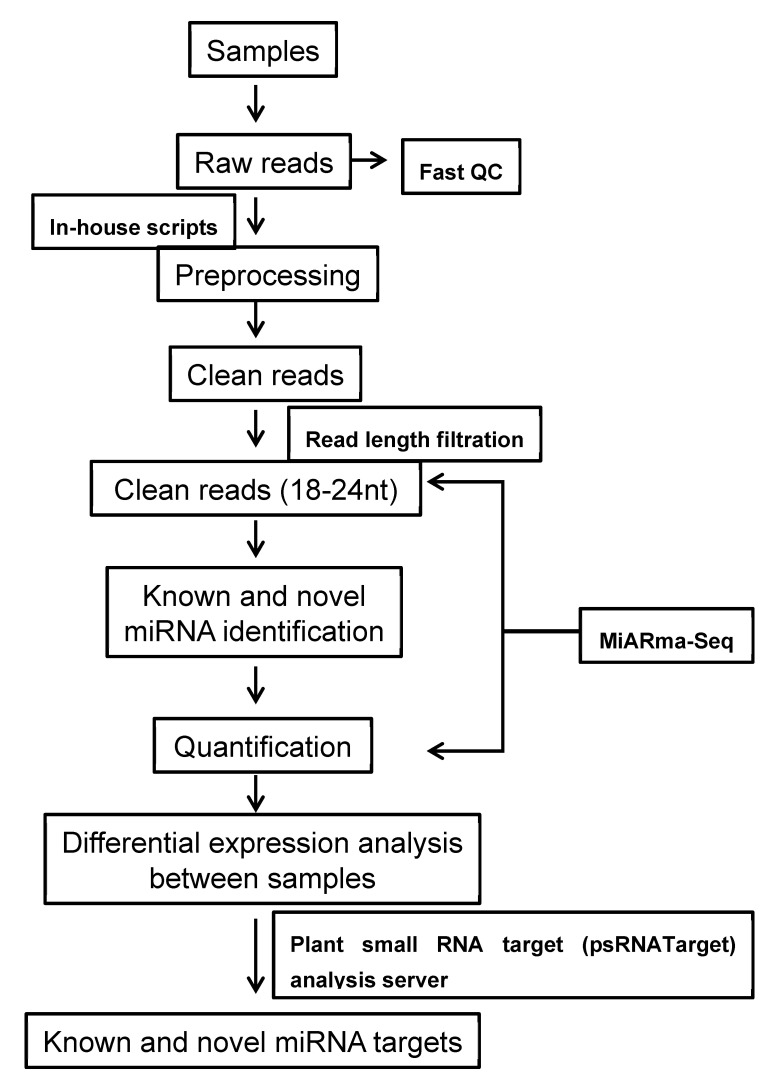
Bioinformatics workflow followed for analysis of the miRNAs. QC: quality check.

The workflow of bioinformatics used for miRNA analysis is given in Figure 9. The raw data was subjected to the processing and elimination of adapter sequences, low-quality reads, and junk reads to obtain clean reads. The clean reads were mapped to the reference rice genome *O. sativa* Nipponbare using bowtie [61]. The obtained clean reads were used for the identification of known miRNAs and prediction of novel miRNAs using miARma-Seq [62], a suite designed to study mRNAs, miRNAs, and circRNAs. Known miRNAs were mapped and quantified using the known miRNA identification pipeline from miARma-Seq, and novel miRNAs were identified and quantified using miRDeep2 of miARma-Seq. The criteria followed for the validation of novel miRNA predictions are as follows: (a) mismatched residues should be no more than 4 between miRNA* and miRNA, (b) there should not be any internal bulge and major loop between miRNA* and miRNA, and (c) the predicted secondary structure should follow the MFEI (minimal folding free energy index). The secondary structures were predicted and assessed using the RNAlifold (http://rna.tbi.univie.ac.at/cgi-bin/RNAWebSuite/RNAalifold.cgi) and Mfold (http://unafold.rna.albany.edu/?q=mfold) web servers. The target genes of miRNAs were predicted using the online tool psRNA target (http://plantgrn.noble.org/psRNATarget/) [63,64]. The criteria used for miRNA target prediction were (1) accepting two mismatches in the seed region of the miRNA, (2) additional acceptance of three mismatches between the 12 and 22 positions without any gaps, (3) length of complementarity score (hspsize): 19, (4) range of central mismatch leading to translation inhibition: 10–11 nt, and (5) a maximum exception value of 0.5 for the extending gap penalty and G:U pair penalty [64].

### 4.4. RT-qPCR Analysis of miRNAs and Target Genes

Small RNA was isolated using mir-Vana™ miRNA isolation kit (Ambion, Carlsbad, CA, USA) according to the manufacturer’s instructions. The isolated miRNAs were normalized and subjected to reverse transcription (RT) using the miScript II RT kit according to the instructions given by the manufacturer (Qiagen, Hilden, Germany). The obtained cDNA was used for qPCR as a template. miScript SYBR Green PCR kit (Qiagen) was used for setting up the qPCR reaction. Apart from the kit components, template cDNA and forward primer specific to miRNAs (Supplementary Table S10) were added in the qPCR reaction mix. U6 was used as the internal reference gene for normalization [65]. The qPCR reaction was carried out in Roche Light Cycler II machine, USA. The reaction temperatures and details of the miRNA quantification procedure are described in our previous reports [16,66]. For the expression analysis of the target genes, mRNAs were isolated from the same sample and column (used for small RNA isolation) using the mir-Vana™ miRNA isolation kit. Long and small RNAs can be isolated from the same preparation using this kit by adjusting the ethanol concentration. The normalized RNA was used for the synthesis of cDNA using the Improm-II reverse transcription system (Promega, Madison, WI, USA). The qPCR was performed using SYBR Premix Ex-Taq (Takara, Kusatsu, Japan) in a Roche Light Cycler II machine, USA. The internal reference for the normalization was *OsActin1* [67]. The qPCR primers of the target genes are given in Supplementary Table S10. The reactions were run in duplicate. Expression of miRNAs and target genes was analyzed in three biological replicates. Melt curve analysis followed by agarose gel electrophoresis was used to ascertain the specificity of the amplification. The relative expression levels were quantified using the comparative threshold cycle (C_T_) method. The details of the methods followed for the fold difference calculation using 2^−ΔΔCT^ and the standard error are described in our previous report [16].

## 5. Conclusions

MicroRNAs confer adaptive changes in the host plant during abiotic and biotic stresses by regulating the transcription factors and key genes. These small RNAs can either target resistance genes or directly regulate defense responses to impart plant immunity against pathogens. The promising resistance or susceptibility-associated miRNAs identified in this study need to be further characterized by (1) allele mining of pri-miRNAs genes/promoters from diverse rice cultivars and wild rice species; (2) overexpression/silencing assays to determine the positive or negative regulation of the host immunity; (3) the movement of these miRNAs between rice and *R. solani*; (4) the dynamics of miRNAs expression and accumulation during the recognition and progression of the disease initiation and establishment; and (5) deciphering the gene regulation network pathways of potential miRNAs such as Osa-miR397b, Osa-miR169a, and miR398b during Rice-*R. solani* interactions. More intensive and consistent research efforts in this area will facilitate the better understanding of the gene regulation and molecular processes during sheath blight disease caused by *R. solani* in rice and the identification of alternate genetic elements for the development of resistance against this invincible pathogen.

## Figures and Tables

**Table 1:** Summary of the small RNA sequencing and analysis of control and *Rhizoctonia solani*-infected rice genotypes.

Sample Name	Total Number of Reads	Clean Reads before Data Filtering	Q30 (%)	Clean Reads after Data Filtering	Clean Reads (18–24 nt)	Identified Known miRNAs	Identified Novel miRNAs
***R. solani*-infected samples**							
TN1	17,292,908	17,277,169	96.65	16,998,190	1,209,465	89	7
BPT5204	19,838,709	19,817,284	96.7	19,603,829	3,442,243	282	8
N22	12,331,370	12,318,143	96.45	12,028,969	1,563,885	24	9
Vandana	15,584,456	15,566,007	96.42	15,179,461	1,082,338	278	21
Tetep	16,772,072	16,754,325	96.64	16,346,243	2,601,317	161	9
Pankaj	14,666,490	14,652,163	96.66	14,207,919	1,023,439	147	4
**Control samples**							
TN1	18,776,171	18,757,059	96.58	18,058,533	822,299	95	7
BPT5204	17,754,335	17,733,540	95.66	17,107,679	1,202,770	104	5
N22	16,568,740	16,549,626	96.38	15,910,033	1,397,952	24	2
Vandana	15,698,991	15,682,752	96.48	14,906,413	1,062,798	154	6
Tetep	15,304,171	15,283,850	96.11	14,364,145	986,089	43	2
Pankaj	16,132,109	16,114,741	96.57	15,524,879	851,888	128	5

## Data Availability

All data generated or analyzed during this study are included in this published article (and its Supplementary Information Files). The sequence data is available on request.

## Supplementary Materials

The supplementary materials can be found at https://www.mdpi.com/1422-0067/21/21/7974/s1, Table S1: List of expressed miRNAs in different small RNA libraries of control and *R. solani*-infected samples. Table S2: Common and exclusive miRNAs expressed in *R. solani*-infected six rice genotypes. Table S3: Common and exclusive miRNAs expressed in the control tissue of six rice genotypes. Table S4: List of miRNA families expressed in 12 small RNA sequencing libraries. Table S5: Predicted novel miRNAs and their characteristics. Table S6: Pair-wise comparison of miRNAs expression in six rice genotypes under control and *R. solani*-infected conditions. Table S7: Common and exclusive miRNAs in susceptible rice genotypes. These miRNAs are preferentially expressed under *R. solani* infection. Table S8: Common and exclusive miRNAs in resistant rice genotypes. These miRNAs are preferentially expressed under *R. solani* infection. Table S9: Common and exclusive miRNAs among the susceptible and resistant rice genotypes. These miRNAs are preferentially expressed under *R. solani* infection. Table S10: Details of predicted target genes of miRNAs, and the list of primers used in this study. Table S11: Details of wild rice accessions used in this study and their disease score against *R. Solani*. Table S12: List of QTLs used for mapping of the miRNAs. Table S13: Biological functions of 14 miRNAs utilized for RT-qPCR analysis.
