# Supplementary material for: A Comprehensive Analysis of MicroRNAs Expressed in Susceptible and Resistant Rice Cultivars during Rhizoctonia solani AG1-IA Infection Causing Sheath Blight Disease
Source: Int J Mol Sci. 2020 Oct 27;21(21):7974. doi: 10.3390/ijms21217974 (PMC7662745; doi:10.3390/ijms21217974)
Supplement: Supplementary file 1 [file ijms-21-07974-s001.zip › Suppl files/Table S5.docx]

| **S.No** | **miRNA ID** | **Estimated probability that the miRNA candidate is a true positive** | **Consensus mature**  **miRNA sequence (5’-3’)** | **Length (nt)** | **Precursor length** | **Precursor coordinate** |
| --- | --- | --- | --- | --- | --- | --- |
|  | **RS1** |  |  |  |  |  |
| 1 | 2752 | 75 +/- 9%, | uaguuuuuucuaccacacuuu | 21 | 45 | Chr2:14279609..14279654:- |
| 2 | 3555 | 75 +/- 9% | uugucauaguuacucugauagg | 22 | 76 | Chr3:16942099..16942175:+ |
| 3 | 200 | 57 +/- 9% | acaauguaucuggauaugagacgu | 24 | 58 | Chr1:7927966..7928024:+ |
| 4 | 13746 | 57 +/- 9% | ccucgccggcgcgcgugcucaccc | 24 | 49 | Chr12:19066704..19066753:+ |
| 5 | 3359 | 57 +/- 9% | uucagauucauaguauugug | 20 | 49 | Chr3:7666006..7666055:+ |
| 6 | 4694 | 57 +/- 9%, | acuagguuuguuuauuuugggac | 23 | 65 | Chr3:33572263..33572328:- |
| 7 | 6507 | 57 +/- 9% | uagauucguaguacuaagaugugu | 24 | 60 | Chr5:17566940..17567000:+ |
|  | **RS2** |  |  |  |  |  |
| 8 | 2847 | 67 +/- 8% | auuuguuguauuagggaaugucuc | 24 | 65 | Chr2:10873083..10873148:+ |
| 9 | 13301 | 67 +/- 8% | aaaaugcauggagaauuauggu | 22 | 86 | Chr7:16338726..16338812:- |
| 10 | 8607 | 64 +/- 7% | guuaauuuuguagacggucccccu | 24 | 77 | Chr4:25939400..25939477:- |
| 11 | 562 | 43 +/- 8% | aaaaccuaggacuggauaggacgu | 24 | 87 | Chr1:20316627..20316714:+ |
| 12 | 7446 | 43 +/- 8% | caagucauucuaguauuucuc | 21 | 91 | Chr4:21966235..21966326:+ |
| 13 | 20937 | 43 +/- 8% | gccuuugucauugauauguggacc | 24 | 65 | Chr12:23312503..23312568:- |
| 14 | 9838 | 43 +/- 8% | uaagucauucuaguauuuccc | 21 | 88 | Chr5:7061540..7061628:- |
| 15 | 20176 | 24 +/- 8% | uuguuuugcacuguagaggu | 20 | 70 | Chr12:25664732..25664802:+ |
|  | **RS3** |  |  |  |  |  |
| 16 | 1225 | 39 +/- 20% | auucuaguacuaugaaucugaac | 23 | 56 | Chr1:2955726..2955782:- |
| 17 | 15509 | 16 +/- 16% | gacauggucacugauauguggauc | 24 | 61 | Chr10:21763869..21763930:- |
| 18 | 11000 | 16 +/- 16% | uaucagaauagugggugccuc | 21 | 84 | Chr7:137880..137964:- |
| 19 | 10261 | 16 +/- 16% | aacuuuuguaugugaauauggac | 23 | 57 | Chr6:27739780..27739837:- |
| 20 | 2536 | 16 +/- 16% | ucaacuucuagcuacgaaccugg | 23 | 61 | Chr2:15842982..15843043:+ |
| 21 | 14459 | 16 +/- 16% | agauucguaguacuaggauguguc | 24 | 62 | Chr10:15104787..15104849:+ |
| 22 | 6388 | 18 +/- 10% | agauucguaguauuagaauauguc | 24 | 62 | Chr4:18608059..18608121:+ |
| 23 | 11555 | 18 +/- 10% | acuucugacuauguaucuggacau | 24 | 68 | Chr7:28322190..28322258:- |
| 24 | 5459 | 18 +/- 10% | auuuuugcaugcggucccuua | 21 | 89 | Chr3:22813337..22813426:- |
|  | **RS4** |  |  |  |  |  |
| 25 | 8364 | 42 +/- 21% | acuuugcucuaccaaaaaauuggu | 24 | 81 | Chr5:4281664..4281745:+ |
| 26 | 11492 | 3 +/- 5% | aucauuuccgaucguugaauc | 21 | 69 | Chr7:3504185..3504254:+ |
| 27 | 11245 | 3 +/- 5% | ucuuguggcaagaacugagu | 20 | 58 | Chr6:27238005..27238063:- |
| 28 | 5299 | 3 +/- 5% | uaucuagauuuguaguaaacgugu | 24 | 57 | Chr3:33558251..33558308:+ |
|  | **RS5** |  |  |  |  |  |
| 29 | 35741 | 40 +/- 17% | uuaaacucggcucaaucug | 19 | 44 | Chr12:13410140..13410184:- |
| 30 | 26380 | 32 +/- 17% | caaaauuuuccaugcacuucg | 21 | 85 | Chr8:22294081..22294166:- |
| 31 | 33122 | 37 +/- 16% | caaaauuuuccaugcacuucg | 21 | 85 | Chr11:10450637..10450722:- |
| 32 | 27700 | 37 +/- 16% | uuaacgaaucuagacacauuu | 21 | 51 | Chr9:16785428..16785479:+ |
| 33 | 27060 | 29 +/- 15% | gacauaucuaagacaaugaacuu | 23 | 60 | Chr9:3969750..3969810:+ |
| 34 | 3990 | 2 +/- 4% | cuugucugaugaugugcagagc | 22 | 80 | Chr1:35374578..35374658:- |
| 35 | 32033 | 0 +/- 1% | uuucugauuguuggaucuagcu | 22 | 66 | Chr11:14010041..14010107:+ |
| 36 | 6210 | 0 +/- 1% | ccuaggacaauguaucuggacaug | 24 | 86 | Chr2:35291033..35291119:+ |
| 37 | 17401 | 0 +/- 1% | uucuaguacaauaaaucuggacu | 23 | 53 | Chr5:23825288..23825341:- |
| 38 | 3024 | 0 +/- 1% | aauggacugcacguguaaaugagc | 24 | 92 | Chr1:15442278..15442370:- |
| 39 | 35847 | 0 +/- 1% | gaugugacauauucuaguacuaug | 24 | 74 | Chr12:17784126..17784200:- |
| 40 | 31556 | 0 +/- 1% | aucuuguggauguaucugaacaug | 24 | 53 | Chr11:2839795..2839848:+ |
| 41 | 3387 | 0 +/- 1% | aauauauuguaaucuaggaugggu | 24 | 69 | Chr1:25224364..25224433:- |
| 42 | 12640 | 0 +/- 1% | uucugcaugucaucaggca | 19 | 80 | Chr4:18450607..18450687:+ |
| 43 | 11996 | 0 +/- 1% | guuuccgaucguuggauauaaca | 23 | 63 | Chr4:2913898..2913961:+ |
| 44 | 12000 | 0 +/- 1% | uuuccgaucguuagaucuagcu | 22 | 65 | Chr4:3142734..3142799:+ |
| 45 | 23734 | 0 +/- 1% | cggugcagucgacugcagaggauc | 24 | 67 | Chr7:24923111..24923178:- |
| 46 | 4992 | 0 +/- 1% | cguuccaucuaggaugaagacgu | 23 | 90 | Chr2:11602463..11602553:+ |
| 47 | 11264 | 0 +/- 1% | accguucuguagucgaagaggcuc | 24 | 51 | Chr3:26394147..26394198:- |
| 48 | 32462 | 0 +/- 1% | uuuccgaucguuagaucuagcu | 22 | 65 | Chr11:25091893..25091958:+ |
| 49 | 4622 | 0 +/- 1% | acuuugucguagaauauaag | 20 | 72 | Chr2:3692785..3692857:+ |
|  | **RS6** |  |  |  |  |  |
| 50 | 5272 | 62 +/- 15% | ucuuaucuaaugauccaggacgu | 23 | 42 | Chr4:9175197..9175239:+ |
| 51 | 13246 | 67 +/- 12% | auguccggauucauuguacuaug | 23 | 50 | Chr10:18002429..18002479:- |
| 52 | 13395 | 52 +/- 10% | agggacauauaucucucugacgug | 24 | 74 | Chr10:22114197..22114271:- |
| 53 | 8960 | 52 +/- 10% | ccgaucuuguggcaagaacua | 21 | 66 | Chr7:139778..139844:+ |
| 54 | 5917 | 32 +/- 8% | aucuggacuguuuggcauagcucc | 24 | 79 | Chr4:7876502..7876581:- |
| 55 | 242 | 32 +/- 8% | accuaguacaaugaaucuggaca | 23 | 54 | Chr1:8701838..8701892:+ |
| 56 | 6835 | 32 +/- 8% | auaaauguagaaaauguuagaaug | 24 | 90 | Chr5:7675390..7675480:+ |
| 57 | 1255 | 32 +/- 8% | agauuuauguuggaugugacacau | 24 | 76 | Chr1:7611876..7611952:- |
| 58 | 7440 | 32 +/- 8% | acuuccggcuguagauuagcccuc | 24 | 86 | Chr5:17707677..17707763:- |
|  | **RS7** |  |  |  |  |  |
| 59 | 997 | 35 +/- 15% | uuuuuuguuggaugugacauc | 21 | 76 | Chr1:4626758..4626834:- |
| 60 | 2744 | 35 +/- 15% | uggacaagauuuuuuacaugguug | 24 | 60 | Chr2:14944712..14944772:- |
| 61 | 12928 | 21 +/- 11% | uuuuaguacuaugaaucuggaca | 23 | 89 | Chr11:24798353..24798442:+ |
| 62 | 531 | 21 +/- 11% | aaaaagucuuauaaccugaa | 20 | 90 | Chr1:30603412..30603502:+ |
| 63 | 5879 | 21 +/- 11% | aaaaucaaccuaguaucggaugug | 24 | 91 | Chr4:25638834..25638925:- |
| 64 | 10908 | 21 +/- 11% | agauauagaucuguagcuaacugc | 24 | 98 | Chr9:22399111..22399209:+ |
| 65 | 7789 | 21 +/- 11% | gguaauggaacugcagaagau | 21 | 45 | Chr6:4849265..4849310:- |
|  | **RS8** |  |  |  |  |  |
| 66 | 10901 | 68 +/- 11% | auucuaauacaacaaaucuggac | 23 | 56 | Chr9:8584773..8584829:- |
| 67 | 14342 | 67 +/- 10% | aaggaucaauguaaucgaacu | 21 | 43 | Chr12:4533240..4533283:- |
| 68 | 2120 | 57 +/- 9% | accuaguacaaugaaucuggaca | 23 | 48 | Chr2:16541653..16541701:+ |
| 69 | 10532 | 36 +/- 10% | aucacgaaaacagggauucugg | 22 | 55 | Chr9:4729929..4729984:+ |
| 70 | 7215 | 36 +/- 10% | ugcuaacuguaagcucaagau | 21 | 51 | Chr5:28191725..28191776:- |
|  | **RS9** |  |  |  |  |  |
| 71 | 6228 | 45 +/- 11% | uccuaguacuaugaauuuggac | 22 | 53 | Chr7:4012952..4013005:- |
| 72 | 3724 | 45 +/- 11% | auauaguauuaugaauuuggac | 22 | 51 | Chr4:23318446..23318497:+ |
|  | **RS10** |  |  |  |  |  |
| 73 | 2557 | 49 +/- 19% | aaaaaggauugaguuauuucg | 21 | 55 | Chr2:6170931..6170986:- |
| 74 | 7670 | 46 +/- 12% | auguccggauucauuguacuaug | 23 | 50 | Chr6:4577000..4577050:- |
| 75 | 12206 | 37 +/- 10% | agggacuuauguuuuuaugagagg | 24 | 51 | Chr10:17498948..17498999:- |
| 76 | 4046 | 37 +/- 10% | uauauauauaugcugugugcuug | 23 | 52 | Chr3:7965475..7965527:- |
| 77 | 11526 | 37 +/- 10% | aaaauauauuggauuuuagaugg | 23 | 59 | Chr10:20183165..20183224:+ |
|  | **RS11** |  |  |  |  |  |
| 78 | 1569 | 48 +/- 19% | aguaguaaaauaugcugacaugg | 23 | 68 | Chr1:5087676..5087744:- |
| 79 | 16384 | 66 +/- 11% | ggccucgcggcgcccggcaccc | 22 | 50 | Chr9:11315..11365:+ |
| 80 | 19636 | 69 +/- 9% | uccuaguacgacgaaucuggac | 22 | 47 | Chr11:849144..849191:+ |
| 81 | 12138 | 69 +/- 9%, | uccuugucugaugaugugcagagc | 24 | 82 | Chr6:429810..429892:- |
| 82 | 17471 | 52 +/- 9%, | agauucguaguacuagaauauguc | 24 | 80 | Chr9:14806836..14806916:- |
| 83 | 11523 | 52 +/- 9%,-, | gugagacgaaucuuuugagacua | 23 | 107 | Chr6:10488722..10488829:+ |
|  | **RS12** |  |  |  |  |  |
| 84 | 2914 | 38 +/- 20% | uauauauauaugcugugugcuug | 23 | 51 | Chr3:7965475..7965526:- |
| 85 | 3286 | 17 +/- 12% | uuccgaucguuagaucuagcuggg | 24 | 59 | Chr4:3142735..3142794:+ |

***Infected samples (RS1-TN1, RS2- BPT5204, RS3-N22, RS4- Vandana, RS5- Tetep, RS6- Pankaj), Control samples (RS7-TN1, RS8-BPT5204, RS9-N22, RS10-Vandana, RS11- Tetep, RS12- Pankaj)***

**RS1**

Chr2_2752,210.2,75 +/- 9%,413,400,0,13,uaguuuuuucuaccacacuuu,aagugugguagaaaaaacu,aagugugguagaaaaaacuacauauaguuuuuucuaccacacuuu,Chr2:14279609..14279654:-

Chr3_3555,23.2,75 +/- 9%,49,47,0,2,uugucauaguuacucugauagg,cuacaugaugaugauauccaaa,cuacaugaugaugauauccaaaaagcuauggauucauuccaaaugaggcuuuuauugucauaguuacucugauagg,Chr3:16942099..16942175:+

Chr1_200,1.5,57 +/- 9%,25,25,0,0,acaauguaucuggauaugagacgu,gucucauguccagauacauugucc,acaauguaucuggauaugagacguaucuagauaugucucauguccagauacauugucc,Chr1:7927966..7928024:+

Chr12_13746,1.5,57 +/- 9%,40,39,0,1,ccucgccggcgcgcgugcucaccc,ggcagagcgcgccgccgucgagc,ccucgccggcgcgcgugcucacccuaggcagagcgcgccgccgucgagc,Chr12:19066704..19066753:+

Chr3_3359,1.1,57 +/- 9%,2,1,0,1,uucagauucauaguauugug,cuaguauuauaaaucuggac,cuaguauuauaaaucuggacaaguuucuguucagauucauaguauugug,Chr3:7666006..7666055:+

Chr3_4694,1,57 +/- 9%,130,130,0,0,acuagguuuguuuauuuugggac,uccaaaauaaacaaacguaguau,uccaaaauaaacaaacguaguauagggugugacacaucccguacuagguuuguuuauuuugggac,Chr3:33572263..33572328:-

Chr5_6507,1,57 +/- 9%,17,17,0,0,uagauucguaguacuaagaugugu,acauuauaguacuguaaaucuaga,acauuauaguacuguaaaucuagacagaggcuugccuagauucguaguacuaagaugugu,Chr5:17566940..17567000:+

**RS2**

Chr2_2847,134,67 +/- 8%,-,262,256,0,6,-,-,-,-,-,auuuguuguauuagggaaugucuc,gacauuuccuaguacaacaaaucu,gacauuuccuaguacaacaaaucugucaggagcuuauccagauuuguuguauuagggaaugucuc,Chr2:10873083..10873148:+

Chr7_13301,47.4,67 +/- 8%,-,92,87,3,2,-,-,-,-,-,aaaaugcauggagaauuauggu,auaauuccccaugcauuuugu,aaaaugcauggagaauuauggugucuaggucgguuccguccauggacuacacugaccaauccaccauaauuccccaugcauuuugu,Chr7:16338726..16338812:-

Chr4_8607,2.4,64 +/- 7%,-,5,4,0,1,-,-,-,-,-,guuaauuuuguagacggucccccu,aggggcugucuacaaaauugucau,guuaauuuuguagacggucccccucccagcgauaaaucacacuaucuccgggaaggggcugucuacaaaauugucau,Chr4:25939400..25939477:-

Chr1_562,1.8,43 +/- 8%,-,104,99,5,0,-,-,-,-,-,aaaaccuaggacuggauaggacgu,guccuauccaguccuagguuuuaa,aaaaccuaggacuggauaggacguuucauaguacaaugacucugagucauuguacuaugaaacguccuauccaguccuagguuuuaa,Chr1:20316627..20316714:+

Chr4_7446,1.4,43 +/- 8%,-,976,941,34,1,-,-,-,-,-,caagucauucuaguauuucuc,auacuggaaugacuugcauu,caagucauucuaguauuucucacauucauauugauguuaaugaauuauuaacaucaauaugaaugugagaaauacuggaaugacuugcauu,Chr4:21966235..21966326:+

Chr12_20937,1,43 +/- 8%,-,39,37,0,2,-,-,-,-,-,gccuuugucauugauauguggacc,auuuauuagugacaaaggcaugg,gccuuugucauugauauguggaccugaugugucgucgggcucauuuauuagugacaaaggcaugg,Chr12:23312503..23312568:-

Chr5_9838,1,43 +/- 8%,-,965,960,3,2,-,-,-,-,-,uaagucauucuaguauuuccc,augauagaaugacuuacauugu,uaagucauucuaguauuucccacguauauaugucuagauucauuaacaccaauauaaaugugguaaaugauagaaugacuuacauugu,Chr5:7061540..7061628:-

Chr12_20176,0.8,24 +/- 8%,-,229,229,0,0,-,-,-,-,-,uuguuuugcacuguagaggu,cuauacaaugcaaaacaagg,uuguuuugcacuguagagguaugccacuacagaauggcauauugcuauaccuauacaaugcaaaacaagg,Chr12:25664732..25664802:+

**RS3**

Chr1_1225,7,39 +/- 20%,-,14,12,0,2,auucuaguacuaugaaucugaac,uucagauuuaucguaauagaauau,auucuaguacuaugaaucugaacauacauauguucagauuuaucguaauagaauau,Chr1:2955726..2955782:-

Chr10_15509,1.9,16 +/- 16%,-,4,3,0,1,gacauggucacugauauguggauc,auccauaugucagcgaucauguuc,gacauggucacugauauguggauccgucgauggugggauccauaugucagcgaucauguuc,Chr10:21763869..21763930:-

Chr7_11000,1.7,16 +/- 16%,-,3,2,0,1,uaucagaauagugggugccuc,aggcacccauuauuccgauaaauu,aggcacccauuauuccgauaaauuauguagaaacuugagguagaaccuaguggguguagaauuuaucagaauagugggugccuc,Chr7:137880..137964:-

Chr6_10261,1.4,16 +/- 16%,-,37,37,0,0,aacuuuuguaugugaauauggac,cuauauucauuuacaaaaguugg,aacuuuuguaugugaauauggacauaggcuaugucuauauucauuuacaaaaguugg,Chr6:27739780..27739837:-

Chr2_2536,1.3,16 +/- 16%,-,15,15,0,0,ucaacuucuagcuacgaaccugg,agauuuguagccagaaguugauu,ucaacuucuagcuacgaaccuggauauagguuauguccagauuuguagccagaaguugauu,Chr2:15842982..15843043:+

Chr10_14459,1,16 +/- 16%,-,92,92,0,0,agauucguaguacuaggauguguc,cacauucuaguacaaugaauuuga,cacauucuaguacaaugaauuugaacagagauauguaaagauucguaguacuaggauguguc,Chr10:15104787..15104849:+

Chr4_6388,0.8,18 +/- 10%,-,16,16,0,0,agauucguaguauuagaauauguc,cauaucuagucuuacgaaucugg,cauaucuagucuuacgaaucuggguauacauauguuccagauucguaguauuagaauauguc,Chr4:18608059..18608121:+

Chr7_11555,0.1,18 +/- 10%,-,15,15,0,0,acuucugacuauguaucuggacau,auccaaauauauagccagaaguua,acuucugacuauguaucuggacauaagacauaaguuauaauaguauccaaauauauagccagaaguua,Chr7:28322190..28322258:-

Chr3_5459,0,18 +/- 10%,-,18,18,0,0,auuuuugcaugcggucccuua,agugacccucguguaaaaauag,auuuuugcaugcggucccuuaagagcacuguauagaaaaauaugcuuauuuuuacaugcagccccuaagugacccucguguaaaaauag,Chr3:22813337..22813426:-

**RS4**

Chr5_8364,8.2,42 +/- 21%,-,16,6,5,5,acuuugcucuaccaaaaaauuggu,aauuaauugguaggguagagaacc,acuuugcucuaccaaaaaauugguagugccaaaauuugccuagauuuugguacuaccaauuaauugguaggguagagaacc,Chr5:4281664..4281745:+

Chr7_11492,0.7,3 +/- 5%,-,23,23,0,0,aucauuuccgaucguugaauc,ucuaacgauaagaaaugauuu,aucauuuccgaucguugaaucuaguuggacagaaugcguauuguuagaucuaacgauaagaaaugauuu,Chr7:3504185..3504254:+

Chr6_11245,0.6,3 +/- 5%,-,11,11,0,0,ucuuguggcaagaacugagu,gcgguucuugucacaagacc,ucuuguggcaagaacugaguaguucguuaacucgcuacgcgguucuugucacaagacc,Chr6:27238005..27238063:-

Chr3_5299,0,3 +/- 5%,-,13,13,0,0,uaucuagauuuguaguaaacgugu,acauccuauuacuacgaauuugaucau,acauccuauuacuacgaauuugaucauacucucuaucuagauuuguaguaaacgugu,Chr3:33558251..33558308:+

**RS5**

Chr12_35741,178.6,40 +/- 17%,-,353,337,0,16,uuaaacucggcucaaucug,agauugagccgaguuuaa,uuaaacucggcucaaucugaaaaaagagauugagccgaguuuaa,Chr12:13410140..13410184:-

Chr8_26380,6,32 +/- 17%,-,12,9,1,2,caaaauuuuccaugcacuucg,gaacugcaugggaaauuuuguu,gaacugcaugggaaauuuuguuggauuggucaggguaguccauggacggaaccgaccuagacgccaaaauuuuccaugcacuucg,Chr8:22294081..22294166:-

Chr11_33122,5.5,37 +/- 16%,-,11,9,0,2,caaaauuuuccaugcacuucg,gaagugcauggggaauuuuuuu,gaagugcauggggaauuuuuuuggauuggucaggguaguccaugcacggaaccaaccuagacaccaaaauuuuccaugcacuucg,Chr11:10450637..10450722:-

Chr9_27700,5.4,37 +/- 16%,-,10,5,0,5,uuaacgaaucuagacacauuu,augugucuagauucguuaacaucu,uuaacgaaucuagacacauuuauauaaaugugucuagauucguuaacaucu,Chr9:16785428..16785479:+

Chr9_27060,2.1,29 +/- 15%,-,5,4,0,1,gacauaucuaagacaaugaacuu,uuuauuguccuaggauaugu,gacauaucuaagacaaugaacuuggauauacauguuuaaguuuauuguccuaggauaugu,Chr9:3969750..3969810:+

Chr1_3990,1.3,2 +/- 4%,-,33,33,0,0,cuugucugaugaugugcagagc,ucugcacaucaucaggcaagaa,ucugcacaucaucaggcaagaaaauuauucauucuaagugaaaagaaugauuaaauuucuugucugaugaugugcagagc,Chr1:35374578..35374658:-

Chr11_32033,0.8,0 +/- 1%,-,12,5,0,7,uuucugauuguuggaucuagcu,uucaacgaucggaaaugauuug,uuucugauuguuggaucuagcuacguauaaugugcauuguuagauucaacgaucggaaaugauuug,Chr11:14010041..14010107:+

Chr2_6210,0.7,0 +/- 1%,-,31,23,8,0,ccuaggacaauguaucuggacaug,ugucuagauacauuguucuaggau,ccuaggacaauguaucuggacaugacauucuauccuaagacaauguauuuggacaugagauaugucuagauacauuguucuaggau,Chr2:35291033..35291119:+

Chr5_17401,0.7,0 +/- 1%,-,49,49,0,0,uucuaguacaauaaaucuggacu,uccagauuuauugucuuaggagg,uucuaguacaauaaaucuggacuauuuuuauccagauuuauugucuuaggagg,Chr5:23825288..23825341:-

Chr1_3024,0.7,0 +/- 1%,-,10,9,1,0,aauggacugcacguguaaaugagc,ucauuuacgcgugcaguccauuaa,ucauuuacgcgugcaguccauuaagaggaccacauggaaaaaaucgauuuuuccgugcgguucucuuaaauggacugcacguguaaaugagc,Chr1:15442278..15442370:-

Chr12_35847,0.6,0 +/- 1%,-,29,28,0,1,gaugugacauauucuaguacuaug,cauaguaauagaauguguuauauc,gaugugacauauucuaguacuaugaauccagacauacauauguucagauucauaguaauagaauguguuauauc,Chr12:17784126..17784200:-

Chr11_31556,0.5,0 +/- 1%,-,13,13,0,0,aucuuguggauguaucugaacaug,uguucagauacauucucgggguug,aucuuguggauguaucugaacaugcaucuuguucagauacauucucgggguug,Chr11:2839795..2839848:+

Chr1_3387,0.4,0 +/- 1%,-,10,10,0,0,aauauauuguaaucuaggaugggu,ccauccuagauuacaauauauugg,aauauauuguaaucuaggauggguccaccuaaaaugggauuagacccauccuagauuacaauauauugg,Chr1:25224364..25224433:-

Chr4_12640,0.4,0 +/- 1%,-,20,20,0,0,uucugcaugucaucaggca,ucugaugaaaugcagaguu,uucugcaugucaucaggcaagaaaauaaaucaucccaauugaaaauuaauuuaauuucuugucugaugaaaugcagaguu,Chr4:18450607..18450687:+

Chr4_11996,0.4,0 +/- 1%,-,14,14,0,0,guuuccgaucguuggauauaaca,uuagauccaacgaacggaaacga,guuuccgaucguuggauauaacaauacccaucuuaucccguuagauccaacgaacggaaacga,Chr4:2913898..2913961:+

Chr4_12000,0.3,0 +/- 1%,-,167,162,1,4,uuuccgaucguuagaucuagcu,agauccaacgaucggaaacgauuu,uuuccgaucguuagaucuagcugggcaggauggacauuguuagauccaacgaucggaaacgauuu,Chr4:3142734..3142799:+

Chr7_23734,0.2,0 +/- 1%,-,10,9,0,1,cggugcagucgacugcagaggauc,gaugcucugcagucgacugcacc,gaugcucugcagucgacugcaccgcaugucaauaauaaaagcgcggugcagucgacugcagaggauc,Chr7:24923111..24923178:-

Chr2_4992,0.1,0 +/- 1%,-,10,10,0,0,cguuccaucuaggaugaagacgu,guccucguccuagaugaaacagu,cguuccaucuaggaugaagacguggcauccuauuaauuucgugcgauggcuguuaauaggaugacauguccucguccuagaugaaacagu,Chr2:11602463..11602553:+

Chr3_11264,0.1,0 +/- 1%,-,70,69,0,1,accguucuguagucgaagaggcuc,aguuucaugacuacaagauggcu,aguuucaugacuacaagauggcugacaaccguucuguagucgaagaggcuc,Chr3:26394147..26394198:-

Chr11_32462,0.1,0 +/- 1%,-,109,109,0,0,uuuccgaucguuagaucuagcu,uuagauuuaacgaucagaaaug,uuuccgaucguuagaucuagcuagcugagcaugaugggcauuguuagauuuaacgaucagaaaug,Chr11:25091893..25091958:+

Chr2_4622,0,0 +/- 1%,-,14,14,0,0,acuuugucguagaauauaag,uauuauucugagauggagguag,acuuugucguagaauauaagagauuuuauccauguaacuauaaaaucucuuauuauucugagauggagguag,Chr2:3692785..3692857:+

**RS6**

Chr4_5272,65.1,62 +/- 15%,-,134,133,0,1,ucuuaucuaaugauccaggacgu,aauccugggcgugacgagu,ucuuaucuaaugauccaggacguaauccugggcgugacgagu,Chr4:9175197..9175239:+

Chr10_13246,3.5,67 +/- 12%,-,7,5,0,2,auguccggauucauuguacuaug,auauuauaacgaaucuggauagu,auauuauaacgaaucuggauaguaagcauguccggauucauuguacuaug,Chr10:18002429..18002479:-

Chr10_13395,1.1,52 +/- 10%,-,21,21,0,0,agggacauauaucucucugacgug,ugucagagacgacuuaugucccucu,agggacauauaucucucugacgugugggucuauuggggugagccucacgugucagagacgacuuaugucccucu,Chr10:22114197..22114271:-

Chr7_8960,1,52 +/- 10%,-,18,18,0,0,ccgaucuuguggcaagaacua,guucuugucauaagauccgag,ccgaucuuguggcaagaacuagguaguucguuaacucgcuacacgguucuugucauaagauccgag,Chr7:139778..139844:+

Chr4_5917,0.9,32 +/- 8%,-,23,23,0,0,aucuggacuguuuggcauagcucc,agcugugccaaacggggccugagua,aucuggacuguuuggcauagcuccugcuccagguaaguuggaguuaggagcuggagcugugccaaacggggccugagua,Chr4:7876502..7876581:-

Chr1_242,0.7,32 +/- 8%,-,23,23,0,0,accuaguacaaugaaucuggaca,uccggauucguuguacuaaguag,accuaguacaaugaaucuggacaagggcauauccggauucguuguacuaaguag,Chr1:8701838..8701892:+

Chr5_6835,0.6,32 +/- 8%,-,35,35,0,0,auaaauguagaaaauguuagaaug,uucuagcauuucucacauucauau,uucuagcauuucucacauucauauaguugguaaugaaucuaaacgucuacauucauuaacaacuauauaaauguagaaaauguuagaaug,Chr5:7675390..7675480:+

Chr1_1255,0.4,32 +/- 8%,-,10,9,1,0,agauuuauguuggaugugacacau,gugucauauccaacaaaaaucccu,agauuuauguuggaugugacacauccuauguccagauacguuguacuaggaugugucauauccaacaaaaaucccu,Chr1:7611876..7611952:-

Chr5_7440,0.1,32 +/- 8%,-,11,8,3,0,acuuccggcuguagauuagcccuc,ggguguccaccgggagagc,ggguguccaccgggagagcugacgagcggagcagaaggcgugugcugugcuugucagaggucacuuccggcuguagauuagcccuc,Chr5:17707677..17707763:-

**RS7**

Chr1_997,1.5,35 +/- 15%,-,18,16,2,0,uuuuuuguuggaugugacauc,ugucacauccaaccaaaaaacc,uuuuuuguuggaugugacauccuaguacuaugauccagauucauaguacuaggaugucacauccaaccaaaaaacc,Chr1:4626758..4626834:-

Chr2_2744,1.2,35 +/- 15%,-,18,18,0,0,uggacaagauuuuuuacaugguug,accauguaaaauaucuuguccaaa,accauguaaaauaucuuguccaaacuugacuucguuuggacaagauuuuuuacaugguug,Chr2:14944712..14944772:-

Chr11_12928,0.6,21 +/- 11%,-,14,10,0,4,uuuuaguacuaugaaucuggaca,agauucguaguaauaggauguguc,uuuuaguacuaugaaucuggacaaguuuucuacaaguuuccuaauccagauuagaaaacuuguccagauucguaguaauaggauguguc,Chr11:24798353..24798442:+

Chr1_531,0.2,21 +/- 11%,-,22,21,0,1,aaaaagucuuauaaccugaa,uucagguuauaagacuuu,uucagguuauaagacuuucuaacaaauuguaucuagauucauuaauaauuauauaaauauggauaauguuaaaaagucuuauaaccugaa,Chr1:30603412..30603502:+

Chr4_5879,0.2,21 +/- 11%,-,21,9,12,0,aaaaucaaccuaguaucggaugug,caccugauccuagauucgauuuuua,aaaaucaaccuaguaucggaugugacauauccuagaacuaugaaucuggacauacaagaugugucacaccugauccuagauucgauuuuua,Chr4:25638834..25638925:-

Chr9_10908,0,21 +/- 11%,-,18,8,10,0,agauauagaucuguagcuaacugc,agcggagauauagaucuguagcuaa,agcggagauauagaucuguagcuaacugcagcacggacucuaagacacaauauguguacgauagguggcagcggagauauagaucuguagcuaacugc,Chr9:22399111..22399209:+

Chr6_7789,0,21 +/- 11%,-,5,1,0,4,gguaauggaacugcagaagau,acuuguggcaguucugauacuucu,acuuguggcaguucugauacuucugguaauggaacugcagaagau,Chr6:4849265..4849310:-

**RS8**

Chr9_10901,7.1,68 +/- 11%,-,14,6,0,8,auucuaauacaacaaaucuggac,cuagauucguuguauuagaaugu,auucuaauacaacaaaucuggacauauguauaucuagauucguuguauuagaaugu,Chr9:8584773..8584829:-

Chr12_14342,3.7,67 +/- 10%,-,11,10,0,1,aaggaucaauguaaucgaacu,guuaugguugcuggucuuuu,guuaugguugcuggucuuuuauaaggaucaauguaaucgaacu,Chr12:4533240..4533283:-

Chr2_2120,1,57 +/- 9%,-,19,19,0,0,accuaguacaaugaaucuggaca,uccagauucauuauacuagguua,accuaguacaaugaaucuggacaaguccagauucauuauacuagguua,Chr2:16541653..16541701:+

Chr9_10532,0.4,36 +/- 10%,-,96,96,0,0,aucacgaaaacagggauucugg,agaaucccagcgguuguucgggucg,aucacgaaaacagggauucuggaacggccaagaaucccagcgguuguucgggucg,Chr9:4729929..4729984:+

Chr5_7215,0.2,36 +/- 10%,-,12,11,0,1,ugcuaacuguaagcucaagau,uuuuggacuagcaagaguugcauu,ugcuaacuguaagcucaagauaugugauuuuggacuagcaagaguugcauu,Chr5:28191725..28191776:-

**RS9**

Chr7_6228,0.6,45 +/- 11%,-,15,9,0,6,uccuaguacuaugaauuuggac,ucagauucguaguacuaagau,uccuaguacuaugaauuuggacggauguauauucagauucguaguacuaagau,Chr7:4012952..4013005:-

Chr4_3724,0.2,45 +/- 11%,-,11,9,0,2,auauaguauuaugaauuuggac,guccagauucguaguacuau,auauaguauuaugaauuuggacaugggacuaguccagauucguaguacuau,Chr4:23318446..23318497:+

**RS10**

Chr2_2557,69.6,49 +/- 19%,-,165,146,0,19,aaaaaggauugaguuauuucg,aaccaacuccaauccuuuuacc,aaaaaggauugaguuauuucgagaaauauauuaaaccaacuccaauccuuuuacc,Chr2:6170931..6170986:-

Chr6_7670,1.9,46 +/- 12%,-,4,3,0,1,auguccggauucauuguacuaug,auaguauaacgaaucuggac,auaguauaacgaaucuggacaagaagcauguccggauucauuguacuaug,Chr6:4577000..4577050:-

Chr10_12206,0.9,37 +/- 10%,-,18,18,0,0,agggacuuauguuuuuaugagagg,ucucacaaaaguacaagucccuau,ucucacaaaaguacaagucccuauauuagggacuuauguuuuuaugagagg,Chr10:17498948..17498999:-

Chr3_4046,0.5,37 +/- 10%,-,14,11,0,3,uauauauauaugcugugugcuug,uaggugugcagcauauauauau,uaggugugcagcauauauauauauauauauauauauauaugcugugugcuug,Chr3:7965475..7965527:-

Chr10_11526,0.1,37 +/- 10%,-,12,12,0,0,aaaauauauuggauuuuagaugg,aucuaaauucuuuuauauuuuaa,aaaauauauuggauuuuagauggaugugacacauucaucuaaauucuuuuauauuuuaa,Chr10:20183165..20183224:+

**RS11**

Chr1_1569,6.2,48 +/- 19%,-,44,42,0,2,aguaguaaaauaugcugacaugg,caaguuaguagauaguuuugcucu,caaguuaguagauaguuuugcucucucuuuauuuaaucucuuccaaguaguaaaauaugcugacaugg,Chr1:5087676..5087744:-

Chr9_16384,3,66 +/- 11%,-,5,4,0,1,ggccucgcggcgcccggcaccc,gugccggcgcugccaaggccaccu,ggccucgcggcgcccggcacccaagcgugccggcgcugccaaggccaccu,Chr9:11315..11365:+

Chr11_19636,2.6,69 +/- 9%,-,4,3,0,1,uccuaguacgacgaaucuggac,ucuagauucguugugcuagg,uccuaguacgacgaaucuggaccuauaucuagauucguugugcuagg,Chr11:849144..849191:+

Chr6_12138,2.1,69 +/- 9%,-,4,3,0,1,uccuugucugaugaugugcagagc,uucugcaugucaucaggca,uucugcaugucaucaggcaagaaaauuaauaauccuaaguaaaaaagaauuauuaaauuccuugucugaugaugugcagagc,Chr6:429810..429892:-

Chr9_17471,1.1,52 +/- 9%,-,40,22,0,18,agauucguaguacuagaauauguc,gacguauccuaguacuaugaaucu,gacguauccuaguacuaugaaucuggauauauauauauauauauauauauaugccgagauucguaguacuagaauauguc,Chr9:14806836..14806916:-

Chr6_11523,1,52 +/- 9%,-,46,9,35,2,gugagacgaaucuuuugagacua,uagacuuaaaagauucgucucgcg,gugagacgaaucuuuugagacuaauuaagcugucauuagcacaugugaguuacuguagcacuuauggcuaaucacggacuaauuagacuuaaaagauucgucucgcg,Chr6:10488722..10488829:+

**RS12**

Chr3_2914,2.2,38 +/- 20%,5,4,0,1,uauauauauaugcugugugcuug,aggugugcagcauauauauauau,aggugugcagcauauauauauauauauauauauauauaugcugugugcuug,Chr3:7965475..7965526:-

Chr4_3286,0,17 +/- 12%,11,11,0,0,uuccgaucguuagaucuagcuggg,cauuguuagauccaacgaucggaaac,uuccgaucguuagaucuagcugggcaggauggacauuguuagauccaacgaucggaaac,Chr4:3142735..3142794:+
