# Supplementary material for: A Comprehensive Analysis of MicroRNAs Expressed in Susceptible and Resistant Rice Cultivars during Rhizoctonia solani AG1-IA Infection Causing Sheath Blight Disease
Source: Int J Mol Sci. 2020 Oct 27;21(21):7974. doi: 10.3390/ijms21217974 (PMC7662745; doi:10.3390/ijms21217974)
Supplement: Supplementary file 1 [file ijms-21-07974-s001.zip › Suppl files/Table S11.doc]

| **Accession number** | **Rice species** | **Average plant height (cm)** | **Average lesion height (cm)** | **Average relative lesion height (cm)** | **Disease Score (SES Scale, IRRI, 2013)** |
| --- | --- | --- | --- | --- | --- |
| IC521783 | *Oryza rufipogon* | 141.5 | 33.5 | 23.7 | 3 |
| EC861666 | *O. officinalis* | 138 | 40 | 28.9 | 3 |
| EC861746 | *O. alta* | 140 | 37.5 | 26.8 | 3 |
| EC861741 | *O.latifolia* | 107.5 | 29 | 27.0 | 3 |
| EC861737 | *O. minuta* | 117 | 27.5 | 23.6 | 3 |
| EC861729 | *O. punctata* | 112.5 | 42.5 | 37.8 | 5 |
| EC861795 | *O. glaberrima* | 94 | 40 | 42.6 | 5 |
| TN-1 (susceptible) | *O. sativa* | 100 | 90 | 90.0 | 9 |
| Tetep (tolerant) | *O. sativa* | 144 | 61.8 | 42.9 | 5 |

**Supplementary Table 11:** Phenotyping of wild rice species for sheath blight disease resistance under glasshouse. The plants were inoculated with highly virulent strain of *R. solani* during the wet season of 2019. The observation values represent average of minimum five plants.
