# Supplementary material for: A Comprehensive Analysis of MicroRNAs Expressed in Susceptible and Resistant Rice Cultivars during Rhizoctonia solani AG1-IA Infection Causing Sheath Blight Disease
Source: Int J Mol Sci. 2020 Oct 27;21(21):7974. doi: 10.3390/ijms21217974 (PMC7662745; doi:10.3390/ijms21217974)
Supplement: Supplementary file 1 [file ijms-21-07974-s001.zip › Suppl files/Table S13.doc]

**Supplementary Table 13: Functions of 14 miRNAs utilized for RT-qPCR analysis.**

| **S.No** | **microRNA** | **Function during host-fungal interaction** | **Reference** |
| --- | --- | --- | --- |
| 1 | Osa-miR156d | A significant reduction in osa‐miR156d accumulation occurred in *Magnaporthe oryzae* elicitor‐treated leaves | Campo *et al.,* 2013 |
| 2 | Osa-miR159b | In MIM159 tobacco, transcriptome profiling revealed that genes associated with defense and programmed cell death were strongly activated, including a suite of 22 PATHOGENESIS-RELATED PROTEIN (PR) genes that were 100- to 1,000-fold up-regulated. MIM159 tobacco appeared immune to Phytophthora infection  Responsive to *M. oryzae* or its elicitors | Zheng *et al.,* 2020  Li *et al.,* 2019a |
| 3 | Osa-miR166h-3p | Rice plants with activated miR166k-166h expression showed enhanced resistance to infection by the fungal pathogens *M. oryzae* and *Fusarium fujikuroi*, the causal agents of the rice blast and bakanae disease, respectively. Stronger induction of miR166k-166h expression occurred in resistant but not in susceptible rice cultivars | Salvador-Guirao *et al.,* 2018 |
| 4 | Osa-miR166j-5p | In a knock-down dcl1 mutant, expression of miR166j-5 was down-regulated, whereas, expression of miR166a/b/c/d-3p/f was up-regulated following *M. oryzae* infection | Zhang *et al.*, 2015 |
| 5 | Osa-miR169a | The transgenic lines overexpressing miR169a became hyper-susceptible to different *M. oryzae* strains associated with reduced expression of defense-related genes and lack of hydrogen peroxide accumulation at the infection site | Li *et al*., 2017 |
| 6 | Osa-miR396f-5p | The transgenic lines over-expressing different miR396 isoforms were highly susceptible to *M. oryzae*  osa-miR396f-5p was identified in response to *R. solani* infection  Overexpression of the osa-miR396f precursor in a susceptible rice variety showed enhanced resistance to *Dickeya zeae* | Chandran *et al.,* 2019  Lin *et al.,* 2016  Li *et al*., 2019 |
| 7 | Osa-miR397b | Responsive to *M. oryzae*  Analysis of Arabidopsis transgenics plants overexpressing Ath-miR397 showed that Ath-miR397b could regulate lignin content through modulation of AtLAC4 | Li *et al.,* 2016  Wang *et al*., 2014 |
| 8 | Osa-miR398b | Over-expression of miR398b displayed higher resistance against *M. oryzae* | Li *et al*., 2014 Li *et al.,* 2019b |
| 9 | Osa-miR528-5p | Negative regulator involved in rice immunity against *M. oryzae*  miR528 negatively regulates viral resistance in rice by cleaving L-ascorbate oxidase (AO) messenger RNA | Li et al 2014  Wu *et al*., 2017 |
| 10 | Osa-miR530-5p | osa-miR530-5p was identified in response to *R. solani* infection  Responsive to *M. oryzae* | Lin *et al.,* 2016  Li *et al*., 2016 |
| 11 | Osa-miR820c | Up-regulated in response to elicitors from the *M. oryzae* in rice  Significantly up-regulated by Rice Stripe Virus infection | Campo *et al*., 2013  Lian *et al*., 2016 |
| 12 | Osa-miR1862d | Responsive to *M. oryzae* or its elicitors | Li *et al*., 2019 |
| 13 | Osa-miR1876 | Responsive to *M. oryzae* or its elicitors | Campo *et al.,* 2013 |
| 14 | Osa-miR2878-5p | Negative regulator involved in rice immunity against *M. oryzae* | Li *et al*., 2014 |

**References**

**Campo S, Peris Peris C, Sire C, Moreno AB, Donaire L, Zytnicki M, Notredame C, Llave C. San Segundo B.** 2013. Identification of a novel micro RNA (mi RNA) from rice that targets an alternatively spliced transcript of the N ramp6 (N atural resistance associated macrophage protein 6) gene involved in pathogen resistance. New Phytologist **199**, 212-227.

**Chandran V, Wang H, Gao F, Cao XL, Chen YP, Li GB, Zhu Y, Yang XM, Zhang LL, Zhao ZX, Zhao JH, Wang YG, Li S, Fan J, Li Y, Zhao JQ, Li SQ, Wang WM.** 2019. miR396-OsGRFs Module Balances Growth and Rice Blast Disease-Resistance. Frontiers in Plant Science **9**, 1999.

**Li Y, Jeyakumar JMJ, Feng Q, Zhao ZX, Fan J, Khaskheli MI, Wang WM.** 2019a. The roles of rice microRNAs in rice-Magnaporthe oryzae interaction, Phytopathology Research **1**, 33.

**Li W, Jia Y, Liu F, Wang F, Fan F, Wang J, Zhu J, Xu Y, Zhong W, Yang J.** 2019. Integration analysis of small RNA and degradome sequencing reveals MicroRNAs responsive to Dickeya zeae in resistant rice. International journal of molecular sciences, **20**, 222.

**Li ZY, Xia J, Chen Z, Yu Y, Li QF, Zhang YC, Zhang JP, Wang CY, Zhu XY, Zhang W, Chen YQ.** 2016. Large-scale rewiring of innate immunity circuitry and microRNA regulation during initial rice blast infection. Nature Scientific reports **6**, 1-10.

**Li Y, Lu YG, Shi Y, Wu L, Xu YJ, Huang F, Guo XY, Zhang Y, Fan J, Zhao JQ, Zhang HY.** 2014. Multiple rice microRNAs are involved in immunity against the blast fungus Magnaporthe oryzae. Plant Physiology **164**, 1077–1092.

**Li Y, Cao XL, Zhu Y, Yang XM, Zhang KN, Xiao ZY, Wang H, Zhao JH, Zhang LL, Li GB, Zheng YP.** 2019b. Osa‐miR398b boosts H2O2 production and rice blast disease‐resistance via multiple superoxide dismutases. New Phytologist **222**, 1507-1522.

**Li Y, Zhao SL, Li JL, Hu XH, Wang H, Cao X-L, Xu YJ, Zhao ZX, Xiao ZY, Yang N, Fan J, Huang F, Wang WM.** 2017. Osa-miR169 Negatively Regulates Rice Immunity against the Blast Fungus Magnaporthe oryzae. Frontiers in Plant Science **8**, 2.

**Lian S, Cho WK, Kim SM, Choi H, Kim KH. 2016**. Time-Course Small RNA Profiling Reveals Rice miRNAs and Their Target Genes in Response to Rice Stripe Virus Infection. PLoS ONE **11**, e0162319.

**Lin R, He L, He J, Qin P, Wang Y, Deng Q, Yang X, Li S, Wang S, Wang W, Liu H.** 2016. Comprehensive analysis of microRNA-Seq and target mRNAs of rice sheath blight pathogen provides new insights into pathogenic regulatory mechanisms. DNA Research **23**, 415-425.

**Salvador Guirao R, Hsing YI, San Segundo B.** 2018. The Polycistronic miR166k-166h Positively Regulates Rice Immunity via Post-transcriptional Control of EIN2. Frontiers in Plant Science **9**, 337.

**Wang CY, Zhang S, Yu Y, Luo YC, Liu Q, Ju C, Zhang YC, Qu LH, Lucas WJ, Wang X, Chen YQ.** 2014. MiR397b regulates both lignin content and seed number in Arabidopsis via modulating a laccase involved in lignin biosynthesis. Plant biotechnology journal **12**, 1132-1142.

**Wu J, Yang R, Yang Z, Yao S, Zhao S, Wang Y, Li P, Song X, Jin L, Zhou T, Lan Y.** 2017. ROS accumulation and antiviral defence control by microRNA528 in rice. Nature plants **3**, 1-7.

**Zhang D, Liu M, Tang M, Dong B, Wu D, Zhang Z, Zhou B.** 2015. Repression of microRNA biogenesis by silencing of OsDCL1 activates the basal resistance to Magnaporthe oryzae in rice. Plant Science **237**, 24-32.

**Zheng Z, Wang N, Jalajakumari M, Blackman L, Shen E, Verma S, Wang MB, Millar AA.** 2020. miR159 Represses a Constitutive Pathogen Defense Response in Tobacco, Plant Physiology **182**, 2182-2198.
